# A sulfur-containing nucleoside antibiotic from *Photorhabdus*

**DOI:** 10.1128/mbio.02164-25

**Published:** 2025-10-31

**Authors:** Sangkeun Son, Negar Shahsavari, Thomas Privalsky, Bryson A. Hawkins, Mi-Hyun Lee, Norman Pitt, Akira Iinishi, Raleb Taher, Nikita Gupta, Chandra Sekar Shankar, Jaini Patel, Kim Lewis

**Affiliations:** 1 Department of Biology, Antimicrobial Discovery Center, Northeastern University 1848, Boston, Massachusetts, USA; McMaster University, Hamilton, Ontario, Canada

**Keywords:** natural antimicrobial products, *Photorhabdus*, nucleoside analogs, *Escherichia coli*, thymidine kinase

## Abstract

We report the discovery of 4′-thiothymidine (4′sT), a sulfur-containing nucleoside antibiotic produced by the nematode symbiont *Photorhabdus asymbiotica*. 4′sT exhibits antibacterial activity against *Escherichia coli*, *Stenotrophomonas maltophilia*, and *Klebsiella pneumoniae*. The production of 4′sT was increased in rifampicin-resistant mutants. Heterologous expression of the radical SAM enzyme-containing gene cluster confirmed its biosynthetic origin. Resistant mutants mapped to *tdk,* which encodes thymidine kinase in the thymidine salvage pathway. Mechanistic studies revealed that 4′sT is a prodrug activated via the thymidine salvage pathway and incorporated into DNA, where it inhibits replication and triggers the SOS response. Clinical *E. coli* isolates were resistant, but inhibition of *de novo* thymidylate synthesis with trimethoprim rendered them highly susceptible, indicating that strain-selective activity is governed by differences in thymidine metabolism. These findings highlight the role of metabolic context in determining antibiotic selectivity and demonstrate the utility of rifampicin resistance for assessing silent biosynthetic gene clusters to discover new antimicrobial compounds.

**IMPORTANCE:**

Introducing novel antibiotics is essential to counter the spread of drug-resistant pathogens. Here, we report the discovery of 4′-thiothymidine (4′sT), a nucleoside antibiotic from the nematode symbiont *Photorhabdus asymbiotica*, identified through activation of a silent biosynthetic gene cluster. 4′sT features an unusual 4′-thiosugar moiety. We identified its biosynthetic gene cluster, including a radical SAM enzyme presumably involved in sulfur incorporation. 4′sT exhibits strain-selective activity that is governed by differences in thymidine metabolism rather than variations in the molecular target. These findings expand our knowledge of antibiotics with unusual selective activity.

## INTRODUCTION

Antibiotic resistance continues to pose a serious threat to public health, with more than one million deaths attributed to resistant infections each year (1). Gram-negative pathogens, such as *Escherichia coli*, *Klebsiella pneumoniae*, *Pseudomonas aeruginosa*, and *Acinetobacter baumannii,* are of particular concern due to their resistance to existing antibiotics. According to the World Health Organization, the top three critical-priority threats are all Gram-negative pathogens (2). The limited availability of clinically effective antibiotics against Gram-negative pathogens is largely due to intrinsic resistance mechanisms, including a restrictive outer membrane and active efflux systems, which prevent many antibiotics from reaching their intracellular targets (3).

Most clinically useful antibiotics were discovered between the 1940s and 1960s from soil-derived actinomycetes (4). However, decades of intensive mining of these organisms have resulted in the repeated rediscovery of known scaffolds, limiting their potential to yield new antibiotics (5, 6). Genome sequencing has revealed numerous novel biosynthetic gene clusters (BGCs) in underexplored microorganisms, highlighting the need to expand antibiotic discovery efforts beyond traditional sources (7, 8). Among these underexplored sources, entomopathogenic bacteria, such as *Photorhabdus* and *Xenorhabdus,* have evolved in unique ecological niches shaped by associations with nematodes and insect hosts (9). This dual-host lifestyle, coupled with competition against other bacterial species, imposes intense selective pressures that drive the expansion and diversification of BGCs. Consequently, these bacteria produce a broad array of specialized metabolites distinct from those found in traditional sources (10, 11). A notable example is darobactin, a bicyclic stapled antibiotic produced by *Photorhabdus* (12). Darobactin is the first antibiotic that targets BamA, an essential outer membrane protein in Gram-negative bacteria. Other examples include odilorhabdins, linear polycationic peptides from *Xenorhabdus* that inhibit protein synthesis (13); evybactin and speirobactin, both of which inhibit *Mycobacterium tuberculosis* by targeting DNA gyrase (14, 15); and DHB (3,6-dihydroxy-1,2-benzisoxazole), which disrupts ubiquinone biosynthesis in Gram-negative bacteria (16).

In addition to targeting underexplored organisms, selecting an effective screening strategy is critical for antibiotic discovery. Traditional antibiotic discovery has typically focused on identifying compounds with activity against a target pathogen. While this approach has led to the discovery of many successful antibiotics in the past, it frequently yields antibiotics mechanistically similar to existing ones or broadly toxic compounds that fail to meet the current critical innovation criteria for the development of new antibiotics (new chemical class, novel target, distinct mode of action, and lack of cross-resistance) (17, 18). A differential screening strategy was developed to address these limitations. This method involves parallel testing against multiple bacterial strains to identify compounds with pathogen-selective activity. Such selectivity, which is rarely observed among existing broad-spectrum antibiotics, is indicative of a novel mode of action (19). It also provides the additional benefit of minimizing disruption to the host microbiota and the spread of resistance among non-target organisms (20, 21). In line with these advantages, recent studies have increasingly reported the discovery of selective antibiotics with distinct modes of action (22–31).

This study reports the discovery of 4′-thiothymidine (4′sT), a new sulfur-containing nucleoside antibiotic with selective activity against Gram-negative pathogens. 4′sT is a prodrug that is activated by the thymidine salvage pathway and incorporated into DNA, where it inhibits DNA synthesis. It was discovered through a combination of differential screening and a targeted elicitation strategy. One obvious barrier to antibiotic discovery from microorganisms is that many BGCs remain silent or express their products poorly under standard laboratory conditions. A variety of approaches have been developed to activate these silent BGCs, including co-culture, genetic manipulation, and the use of chemical elicitors (32, 33). In this work, production of 4′sT was induced by selection for rifampicin-resistant mutants that carry a dysregulated RNA polymerase.

## RESULTS

### Identification of 4′sT

To identify compounds selectively active against Gram-negative pathogens, we fractionated culture extracts of *Photorhabdus* and *Xenorhabdus* strains using high-performance liquid chromatography (HPLC) and screened the resulting fractions against *E. coli* and *Staphylococcus aureus*. During the initial screening, a single fraction from *Photorhabdus asymbiotica* KLE11370 exhibited selective antibacterial activity against *E. coli* MG1655 but not *S. aureus* HG003. However, in subsequent cultures grown under the same conditions, this activity was completely lost. Instead, the strain consistently produced high levels of darobactin, a known antibiotic selectively active against Gram-negative bacteria (12). This shift in secondary metabolite production may result from genetic or epigenetic alterations acquired during prolonged laboratory maintenance, or subtle, uncharacterized changes in growth conditions that affect BGC expression (34, 35). Such instability in secondary metabolism is a common obstacle in natural product discovery, particularly when activity depends on cryptic or conditionally expressed BGCs (36–38).

**Fig 1:**
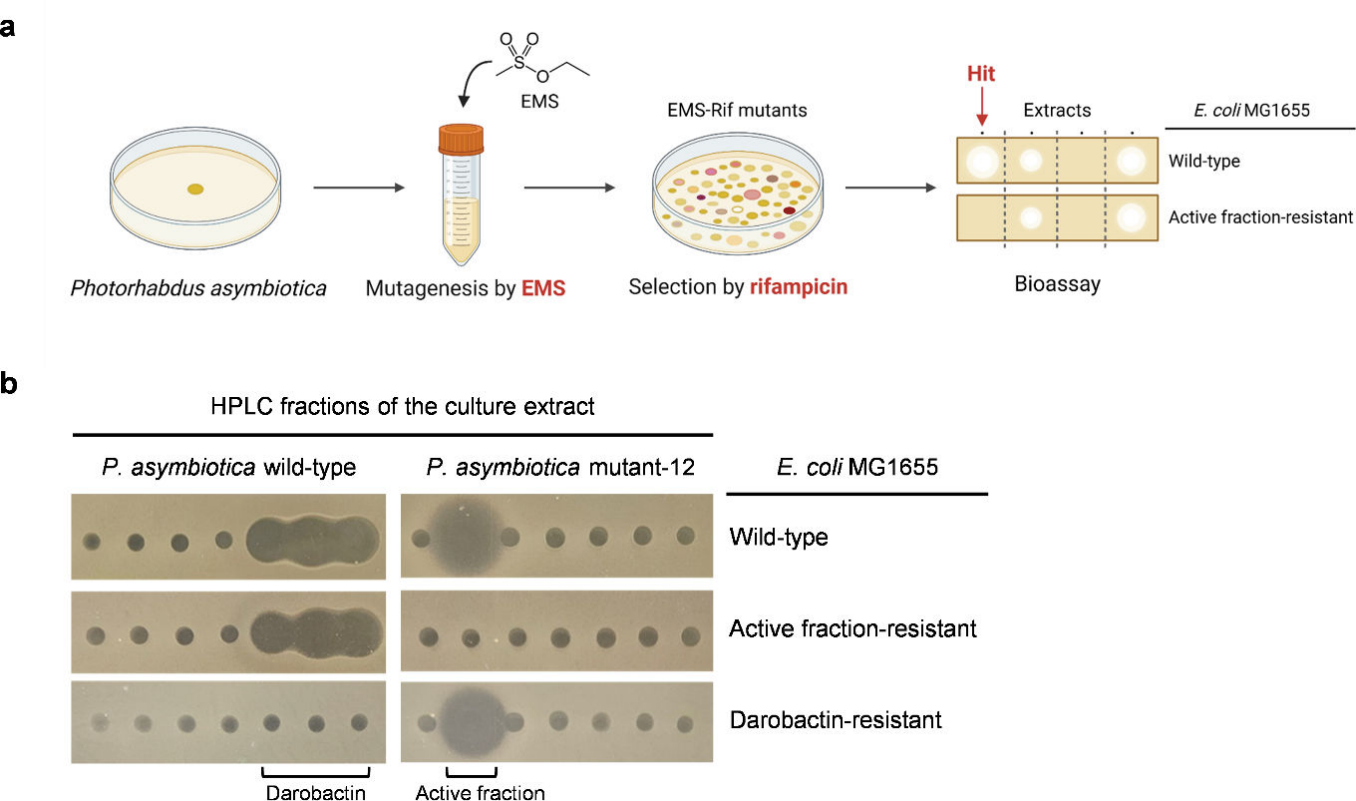
Elicitation of the active compound from *P. asymbiotica* KLE11370 by EMS-Rif strategy. (**a**) Scheme for EMS-Rif strategy for targeted elicitation of the active compound. (**b**) Antimicrobial assay for the HPLC fractions of the culture extracts from *P. asymbiotica* KLE11370 wild-type and EMS-Rif mutant-12. Darobactin-resistant *E. coli* MG1655 carries the triple mutations in *bamA* (T434A, Q445P, and A705T).

Attempts to restore the lost activity by altering media conditions, adding chemical elicitors, or modifying extraction protocols were unsuccessful. We then subjected *P. asymbiotica* KLE11370 to ethyl methanesulfonate (EMS) mutagenesis, which introduces random point mutations, and selected for rifampicin resistance by plating EMS-treated cells on rifampicin-containing agar (Fig. 1a). Rifampicin-resistant mutations in *rpoB*, which encodes the β-subunit of RNA polymerase, have been shown to enhance secondary metabolite production in diverse bacteria by globally altering transcription (39, 40). The resulting mutants (EMS-Rif mutants) were therefore expected to carry *rpoB* mutations. Screening these EMS-Rif mutants for production of the target compound posed two main challenges: (i) *P. asymbiotica* KLE11370 produces darobactin, which also selectively targets Gram-negative bacteria and could lead to false positives in activity-based screening; and (ii) the molecular weight of the target compound was unknown, limiting the utility of liquid chromatography-mass spectrometry (LC-MS)-based detection. To address these issues, we isolated a spontaneous *E. coli* mutant resistant to the original active fraction that contains the target compound by spotting the active fraction onto the lawn of *E. coli*. Extracts from EMS-Rif mutants were then tested against both wild-type and resistant *E. coli* strains; only those that selectively inhibited the wild-type strain were considered potential hits (Fig. S1). This screen yielded 12 EMS-Rif mutants with such selective activity, and one (mutant-12) was selected for scale-up and HPLC fractionation. The active fraction from mutant-12 inhibited wild-type *E. coli* but not the resistant *E. coli* strain, confirming the restored production of the target compound (Fig. 1b). Furthermore, the active fraction showed activity against a darobactin-resistant *E. coli* strain, providing additional evidence that the restored activity was not due to darobactin. Whole-genome sequencing of mutant-12 revealed a non-synonymous mutation in *rpoB* (S531F; TCT → TTT) (Table S1).

### Structure determination

**Fig 2:**
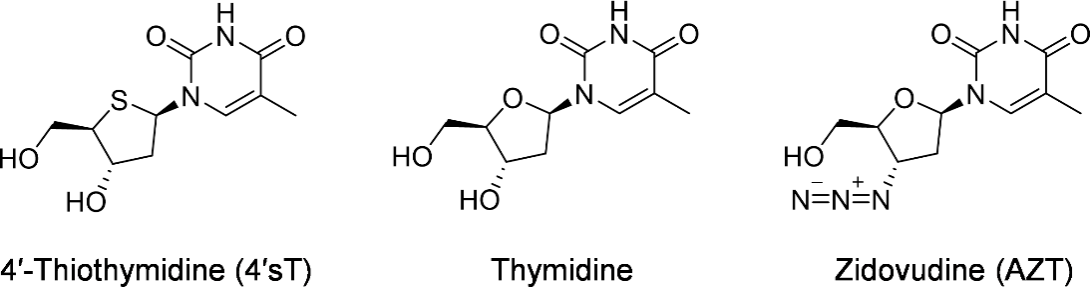
Structures of 4′sT, thymidine, and zidovudine (AZT).

Bioassay-guided purification of the extract from mutant-12 using ion-exchange and reversed-phase chromatography yielded a pure compound. Structural elucidation was carried out using nuclear magnetic resonance (NMR) spectroscopy and high-resolution electrospray ionization mass spectrometry (HRESIMS) (Fig. S2). HRESIMS revealed a protonated molecular ion at m/z 259.0739 [M+H]^+^, consistent with the molecular formula C_10_H_14_N_2_O_4_S (calculated [M+H]^+^ = 259.0747, ∆3.1 ppm), suggesting the presence of a sulfur atom (Fig. S2). Analysis of 1D and 2D NMR data showed signals corresponding to a 2′-deoxyribose unit and a thymine residue (Fig. S2). Comparison with the authentic compound ribothymidine (experimental [M+H]^+^ = 259.0918 m/z, calculated [M+H]^+^ = 259.0925 m/z, ∆2.7 ppm, molecular formula C₁₀H₁₄N₂O₆) revealed that both compounds shared a fragment ion at m/z 127.0499, confirming the presence of an intact thymine moiety and suggesting that the sulfur atom is located in the sugar unit (Fig. S3). The position of the sulfur was determined by the ^1^H NMR chemical shift pattern characteristic of a 4′-thiosugar; specifically, the downfield shift of H_2_-5′ relative to H-4′ matched reported values for synthetic 4′-thionucleosides (41). These data established the structure as 4′sT (Fig. 2). The synthetic standard (purchased from WuXi AppTec) exhibited identical spectroscopic properties and biological activity to the purified compound, confirming the structural assignment (Fig. S2).

### Biosynthetic gene cluster

**Fig 3:**
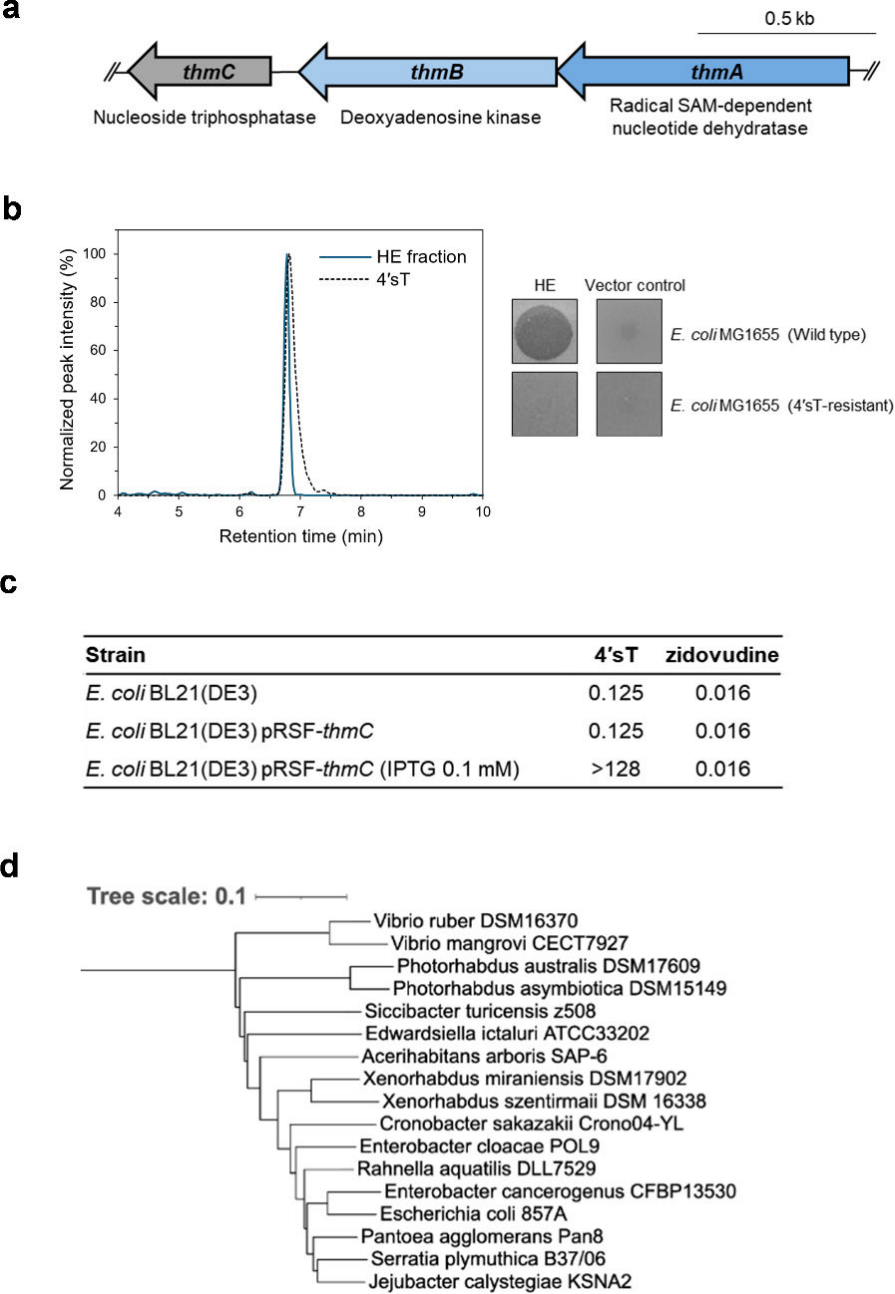
Identification of the 4′sT BGC. (**a**) Organization of the 4′sT BGC. (**b**) Heterologous expression of the 4′sT BGC. Left: extracted ion chromatograms (m/z 259.07) from the active fraction of the heterologous expression (HE) culture and authentic 4′sT standard. Right: antibacterial activity of partially purified extracts from the HE and vector control cultures against *E. coli* MG1655 wild-type and a 4′sT-resistant strain. (**c**) MICs of 4′sT and AZT against *E. coli* BL21(DE3) harboring the *thmC* expression construct. (**d**) Phylogenetic distribution of 4′sT-like BGCs across representative strains. The tree is based on the amino acid sequence of ThmA. Branch lengths and scale bar represent the average number of nucleotide substitutions per base between sequences.

For identification of BGC for 4′sT, we used the genome of *P. asymbiotica* DSM 15149, which was also found to produce 4′sT during continued screening of *Photorhabdus* strains. This strain was selected because its genome is fully annotated, which facilitated downstream analysis. Pairwise OrthoANIu comparison showed 99.93% nucleotide identity between DSM 15149 and *P. asymbiotica* KLE11370, indicating the strains are nearly identical (42). 4′sT has not previously been reported as a natural product. The only natural product incorporating a 4′-thiosugar moiety in the structure is albomycin, a tRNA synthetase inhibitor that contains 4′-thiocytidine (43). At the time of this study, since the mechanism for 4′-thionucleoside formation in albomycin biosynthesis was unknown, all genes from the albomycin BGC were searched for homologs in *P. asymbiotica* DSM 15149 using NCBI tblastn with a maximum E-value threshold of 0.05. The neighboring genomic regions of all homologs found were examined for the presence of additional genes that could be involved in the biosynthesis of a thionucleoside. One candidate BGC, was identified based on the presence of a distant homolog of *abmG* (designated *thmB*, 34.5% amino acid identity, 69% query cover), a nucleoside kinase recently reported to catalyze phosphorylation and dehydration in the formation of the thiofuranose core in albomycin biosynthesis (Fig. 3a; Fig. S4) (44). In *P. asymbiotica*, the homolog of this gene was located adjacent to both a radical SAM enzyme (*thmA*), which could be involved in the synthesis of the 4′-thioribose, as well as a nucleoside triphosphatase (*thmC*) that could either be involved in biosynthesis or provide self-resistance to the product of the BGC (45). It is worth noting that the albomycin BGC also contains two radical SAM enzymes, although neither of these has significant amino acid identity to the one found in *P. asymbiotica*. To narrow down the minimal BGC, *thmA-C* and their flanking genes in *P. asymbiotica* were searched against a collection of 42 *Photorhabdus* and *Xenorhabdus* genomes using cblaster (46). We observed that *thmA-C* co-clustered with each other in organisms where any of them were present, but their surrounding genes differed from *P. asymbiotica* in every other species; the *thmA-C* cluster was found in Fig. S4. In addition, the genes flanking *thmA-C* in *P. asymbiotica* did co-cluster together in other species, although never with *thmA-C*. Based on these observations, we designated *thmA-C* as a candidate minimal BGC for 4′sT.

To validate this, we cloned the three candidate genes *thm*A-C into an IPTG-inducible plasmid (pRSF-*thmA-C*) and expressed them in *E. coli* BL21(DE3). HPLC fractions from induced cultures were active against wild-type *E. coli*, but not against a 4′sT-resistant mutant (Fig. 3b). ESI-MS analysis confirmed the presence of 4′sT in the active fraction, demonstrating that *thmA-C* is the biosynthetic gene cluster for 4′sT. Notably, whereas *E. coli* BL21(DE3) without *thmA-C* induction was susceptible to 4′sT (MIC = 0.125 µg/mL), expression of *thmA-C* conferred resistance (MIC > 128 µg/mL), indicating that a resistance gene is encoded within the cluster. Given the prodrug nature of nucleoside antibiotics, which requires triphosphorylation for activation, we hypothesized that the nucleotide triphosphatase *thmC* might function as the resistance gene. Indeed, expression of *thmC* alone in *E. coli* BL21(DE3) conferred resistance to 4′sT (MIC > 128 µg/mL), whereas susceptibility to the synthetic thymidine analog zidovudine (AZT) was unaffected, indicating that *thmC* specifically mediates resistance to 4′sT (Fig. 3c). To assess the distribution of the 4′sT biosynthetic gene cluster, we searched for homologs of the radical SAM enzyme *thmA* using NCBI blast. Multiple species of γ-proteobacteria contain three adjacent genes homologous to *thmA-C*, suggesting that this family of BGC is conserved across diverse taxa (Fig. 3d). Interestingly, all such organisms, except for *Vibrio*, belong to the order Enterobacterales. Further studies are needed to characterize the structure and function of the encoded compounds in these conserved clusters.

### Potency and spectrum of activity

MIC testing demonstrated potent activity against *E. coli* strains derived from K-12 (MG1655, AB1157, and BW25113), as well as the B strain BL21, with MIC values ranging from 0.0625 to 0.125 µg/mL (Table 1). 4′sT showed bactericidal activity in time-kill assays (Fig. S5). In contrast, no antibacterial activity was observed against clinical *E. coli* isolates (ATCC 25922, ATCC 700928, and ATCC 43888) at concentrations up to 128 µg/mL (Table 1). 4′sT had the same MIC against *E. coli* W0153, a strain carrying a mutation in *lpxC* that increases outer membrane permeability and a deletion of the *tolC* efflux pump gene, as that of MG1655 (47). It also exhibited activity against the Gram-negative pathogens *Stenotrophomonas maltophilia* ATCC 51331 (MIC = 2 µg/mL) and *K. pneumoniae* BAA-1705 (MIC = 8 µg/mL), but not against *S. maltophilia* ATCC 13637, *K. pneumoniae* ESBL JMI 1052654, and *K. pneumoniae* ATCC 700603 (MIC > 128 µg/mL). Additionally, no activity was detected against other Gram-negative species, including *P. aeruginosa*, *A. baumannii*, and *Shigella boydii*, nor against the Gram-positive *S. aureus*. No inhibition was observed against a phylogenetically diverse panel of commensal gut bacteria. Cytotoxicity testing revealed no growth inhibition of human primary lung fibroblasts or mammalian cancer cell lines (HepG2, FaDu, HEK293, and A549) at concentrations up to 128 µg/mL. Taken together, these results indicate that 4′sT exhibits highly potent and narrow-spectrum antibacterial activity with high species and strain selectivity.

**TABLE 1:** MICs of 4′sT

Strain	MIC (μg/mL)
Pathogens	
*Escherichia coli* MG1655	0.0625
*Escherichia coli* AB1157 W0153	0.0625
*Escherichia coli* BW25113	0.125
*Escherichia coli* BW25113 *ΔtolC*	0.125
*Escherichia coli* BL21(DE3)	0.125
*Escherichia coli* ATCC 25922	>128
*Escherichia coli* ATCC 700928	>128
*Escherichia coli* ATCC 43888	>128
*Stenotrophomonas maltophilia* ATCC 51331	2
*Stenotrophomonas maltophilia* ATCC 13637	>128
*Klebsiella pneumoniae* ATCC BAA-1705	8
*Klebsiella pneumoniae* ESBL JMI 1052654	>128
*Klebsiella pneumoniae* ATCC 700603	>128
*Pseudomonas aeruginosa* PAO1	>128
*Acinetobacter baumannii* ATCC 17978	>128
*Yersinia enterocolitica* ATCC 9610	>128
*Citrobacter freundii* ATCC 8090	>128
*Vibrio parahaemolyticus* ATCC 17802	>128
*Enterobacter cloacae* ATCC 13047	>128
*Shigella boydii* ATCC 8700	>128
*Salmonella enterica* ATCC 13076	>128
*Enterococcus faecalis* ATCC 19433	>128
*Staphylococcus aureus* HG003	>128
Gut anaerobes	
*Enterococcus faecalis* KLE 2341	>128
*Clostridium hathewayi* KLE 1709	>128
*Ruminococcus gnavus* KLE 2341	>128
*Veillonella ratti* KLE 2366	64
*Eggerthella lenta* KLE 2234	64
*Bifidobacterium bifidum* KLE 2535	>128
*Bacteroides uniformis* KLE 1601	>128

### Thymidine kinase is essential for the activity of 4′sT

To investigate the mechanism of action of 4′sT, we selected spontaneous resistant mutants by plating *E. coli* MG1655 on agar medium containing 4′sT. Resistant colonies appeared at a relatively high frequency of 9.1 × 10^−6^ on plates supplemented with 128-fold the MIC, suggesting that the resistance arises from null mutations in non-essential genes. Two large resistant mutant colonies exhibiting high-level resistance (MIC > 128 µg/mL) were isolated and subjected to whole-genome sequencing. Both carried mutations in *tdk*, which encodes thymidine kinase—the enzyme responsible for the first phosphorylation step in the thymidine salvage pathway (Table 2). One isolate harbored a missense mutation resulting in a tyrosine-to-aspartate substitution at position 26 (Y26D; TAC → GAC), likely impairing enzymatic function. The other carried an IS1 insertion within the *tdk* coding region, disrupting the open reading frame.

**TABLE 2:** Spontaneous resistant mutants of *E. coli* MG1655 against 4′sT

Colony	4′sT MIC (µg/mL)	Mutation type	Gene	Annotation
RM1	>128	Missense mutation	Thymidine kinase (*tdk*)	Y26D (TAC → GAC)
RM2	>128	Transposon insertion	Thymidine kinase (*tdk*)	IS1 insertion within coding region (213–220 nt)

**Fig 4:**
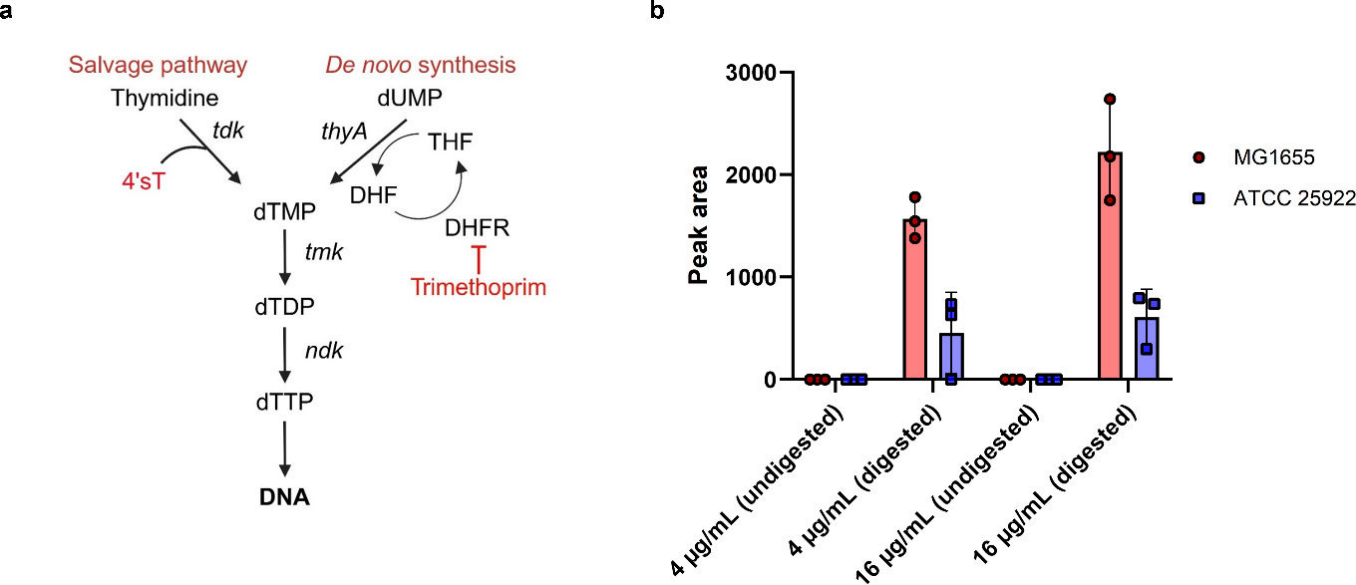
Incorporation of 4′sT into DNA by the thymidine salvage pathway. (**a**) Schematic of the thymidine salvage and *de novo* synthesis pathways. THF, tetrahydrofolate; DHF, dihydrofolate; DHFR, dihydrofolate reductase. (**b**) LC-MS analysis of 4′sT in DNA from *E. coli* MG1655 and ATCC 25922 treated with 4′sT. Genomic DNA was either undigested or digested to single nucleosides prior to analysis. Data represent biological triplicates.

The thymidine salvage pathway allows *E. coli* to import exogenous thymidine and convert it into thymidine triphosphate (dTTP) for DNA replication. In this pathway, thymidine is phosphorylated in three sequential steps: thymidine kinase (*tdk*) catalyzes the initial conversion of thymidine to thymidine monophosphate (dTMP), which is then phosphorylated by thymidylate kinase (*tmk*) to thymidine diphosphate (dTDP), and finally by nucleoside diphosphate kinase (*ndk*) to generate dTTP, which is incorporated into DNA (Fig. 4a) (48). The KEIO collection *tdk* knockout strain (BW25113 ∆*tdk*) showed complete resistance to 4′sT (MIC > 128 µg/mL), confirming that *tdk* is essential for 4′sT activity (49). The absence of spontaneous resistance mutations in *tmk* or *ndk* can be attributed to their essential roles in thymidine metabolism. *tmk* is required for viability, as it catalyzes dTMP phosphorylation in both *de novo* and salvage pathways. While *ndk* is not strictly essential, its loss causes severe genomic instability, likely preventing the emergence of resistant mutants (50). In contrast, *tdk* is dispensable under standard conditions, since cells can generate dTMP *de novo*. The non-essential nature of *tdk* accounts for the relatively high frequency of spontaneous resistance to 4′sT. This also explains the inherent resistance of *tdk*-deficient species, such as *A. baumannii* and *P. aeruginosa*, which are unable to activate 4′sT via the salvage pathway (51). To examine whether nucleoside transport contributes to 4′sT activity, we tested *E. coli* mutants lacking either *nupC* (BW25113 ∆*nupC*) or *nupG* (BW25113 ∆*nupG*). *nupC* encodes a nucleoside:H^+^ symporter specialized for pyrimidine nucleosides, such as thymidine, while *nupG* has broader substrate specificity (52, 53). Deletion of *nupC* led to an eightfold increase in the MIC of 4′sT (MIC = 1 µg/mL), whereas deletion of *nupG* had no effect, indicating that *nupC* is involved in 4′sT uptake.

### 4′sT is incorporated into DNA and inhibits DNA replication

**Fig 5:**
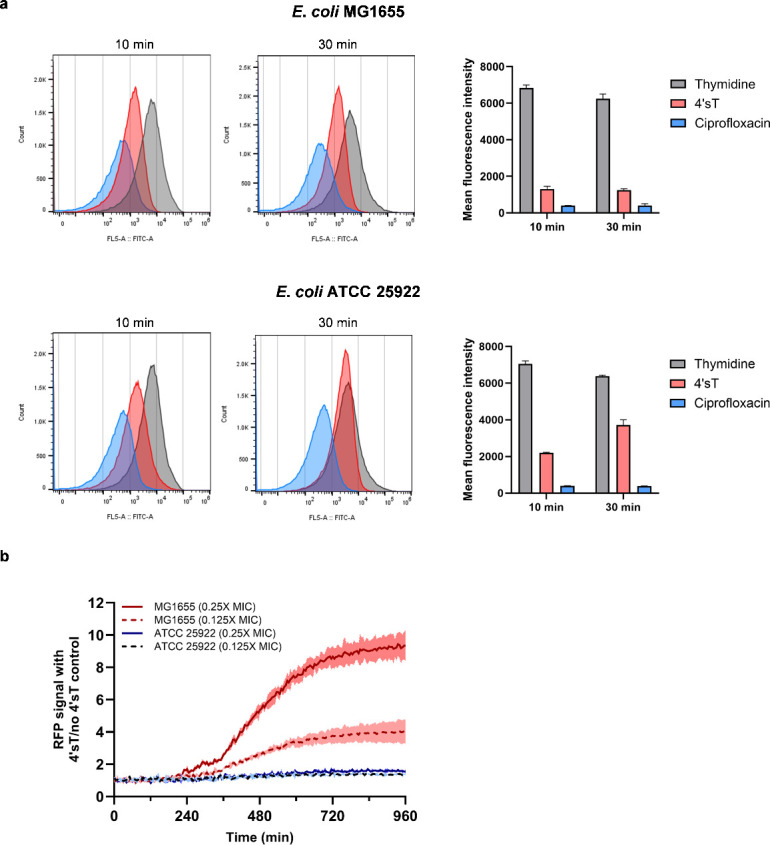
4′sT inhibits DNA replication and induces the SOS response. (**a**) Flow cytometry analysis of EdU incorporation in *E. coli* MG1655 and ATCC 25922. Cells were treated with thymidine (0.25 µg/mL), 4× MIC of 4′sT or ciprofloxacin. Compounds were added for 5 or 25 min, followed by a 5 min pulse with EdU. Histograms show the distribution of fluorescence intensity from a representative replicate. Bar graphs (right) display the mean fluorescence intensity ± s.d. from two biological replicates. (**b**) Time-course analysis of SOS response activation in *E. coli* MG1655 and ATCC 25922 measured by a red fluorescent protein (RFP) reporter gene under control of the *sulA* promoter. Cells were treated with 4′sT at 0.25× or 0.125× MIC against MG1655 (0.0156 and 0.0078 µg/mL, respectively) and RFP fluorescence was measured over time using a microplate reader. RFP signal was normalized to that of untreated controls. Shaded areas represent the mean ± s.d. of three biological replicates.

Because *tdk* is essential for 4′sT activity, we next evaluated whether 4′sT is ultimately incorporated into DNA—the final step of the thymidine salvage pathway. *E. coli* MG1655 cells were treated with 4′sT, followed by genomic DNA extraction, enzymatic digestion, and LC-MS analysis (54). 4′sT was detected in the digested DNA, indicating that it is converted into its triphosphate form (4′sT-TP) and incorporated into the genome (Fig. 4b). To assess the functional consequence of 4′sT incorporation, we examined its effect on DNA replication by measuring the incorporation rate of 5-ethynyl-2′-deoxyuridine (EdU), a thymidine analog. A short (10 min) treatment with 4′sT significantly reduced EdU incorporation, whereas thymidine had no effect, suggesting that 4′sT rapidly disrupts DNA replication (Fig. 5a). This was further supported by the decrease in DNA supercoiling upon 4′sT treatment, which is indicative of replication stress (55) (Fig. S6). In addition, 4′sT triggered a dose- and time-dependent activation of the SOS response as measured by increased expression of a *sulA::rfp* reporter (56) (Fig. 5b). Given that the SOS response is triggered by the accumulation of single-stranded DNA at stalled replication forks, the observed SOS induction suggests that 4′sT impairs DNA replication as its primary mode of action.

The strain-selective activity of 4′sT was puzzling, given that the thymidine salvage pathway is conserved across *E. coli* strains. We performed parallel experiments in *E. coli* ATCC 25922, a clinical isolate resistant to 4′sT. 4′sT was also incorporated into the genomic DNA of ATCC 25922, but at significantly lower levels than in MG1655 (Fig. 4b). DNA replication in ATCC 25922 was initially suppressed following 4′sT treatment but began to recover within 30 min, suggesting that the inhibition was transient and reversible in this strain (Fig. 5a). Unlike in MG1655, 4′sT did not activate the SOS pathway in ATCC 25922 (Fig. 5b). The frequency of antibiotic-resistant mutants did not increase following 4′sT treatment, indicating that error-prone polymerases induced by the SOS pathway were not activated (Fig. S7) (57). These findings indicate that although 4′sT can enter ATCC 25922 cells and be incorporated into DNA to some extent, the strain possesses an intrinsic mechanism to mitigate or bypass its effects on DNA replication without triggering the SOS response.

### Suppression of *de novo* thymidylate synthesis unmasks 4′sT susceptibility in resistant strains

**Fig 6:**
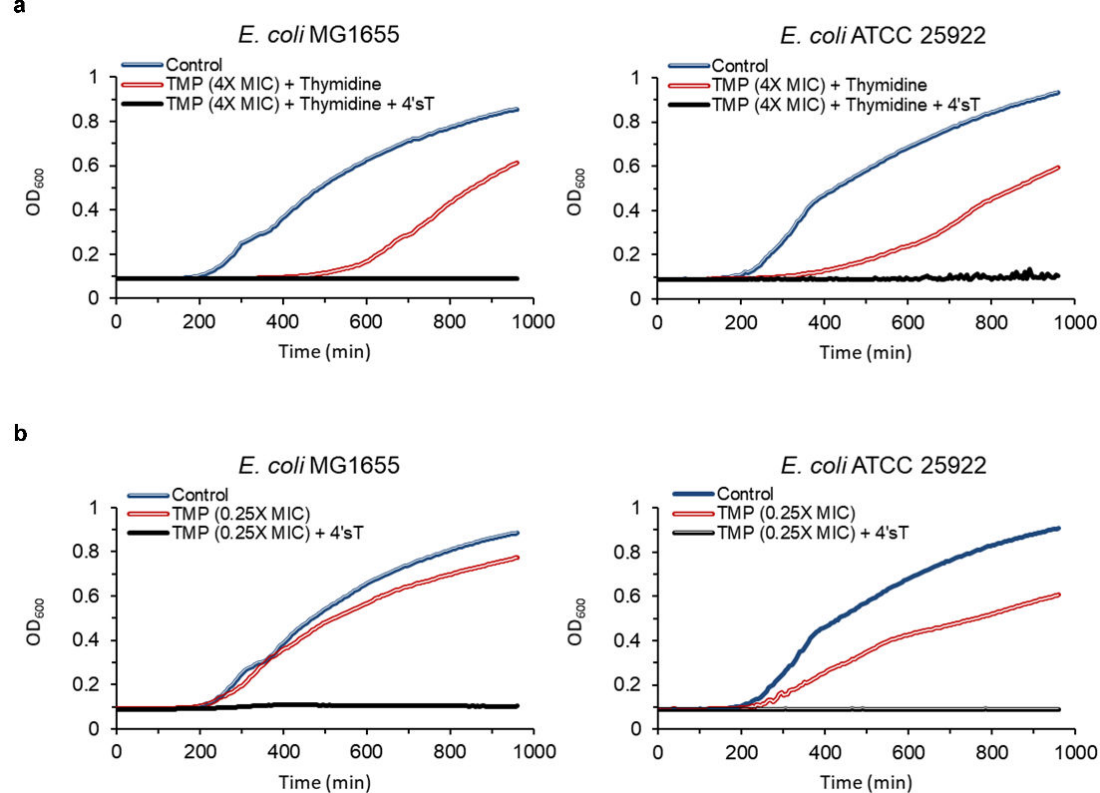
Growth inhibition by 4′sT under conditions of impaired *de novo* thymidylate synthesis by TMP. Growth curves of *E. coli* MG1655 and ATCC 25922 treated with 4′sT in the presence of TMP. (**a**) Cells were treated with TMP at 4× MIC and supplemented with exogenous thymidine (12.5 µg/mL), with or without 4′sT (0.5 µg/mL). (**b**) Cells were treated with TMP at 0.25× MIC with or without 4′sT (0.031 µg/mL). OD600 was measured over time using a microplate reader. Data represent the mean of biological duplicates.

Based on the high sequence similarity of genes involved in the thymidine salvage pathway across *E. coli* strains, we reasoned that substrate selectivity is not the determinant of strain selectivity. We then shifted our focus to the alternative thymidine metabolic route—the *de novo* thymidylate biosynthesis pathway. In *E. coli*, this pathway synthesizes dTMP from deoxyuridine monophosphate via the sequential action of thymidylate synthase (*thyA*) and folate-dependent enzymes, such as dihydrofolate reductase (DHFR) (Fig. 4a) (58). The salvage and *de novo* pathways are coordinately regulated to maintain proper intracellular levels of dTTP for DNA replication. We hypothesized that in resistant strains, the activity of 4′sT is masked by enhanced *de novo* pathway activity, which would reduce the likelihood of 4′sT being incorporated into DNA. To test this hypothesis, we evaluated the activity of 4′sT under conditions in which *de novo* thymidylate synthesis is impaired. Trimethoprim (TMP), a DHFR inhibitor, blocks tetrahydrofolate (THF) production and thereby suppresses dTMP synthesis via the *de novo* pathway (Fig. 4a) (59). TMP treatment suppresses the *de novo* pathway and induces thymidineless death; however, viability is maintained in the presence of exogenous thymidine utilized via the salvage pathway (60). Under these conditions, the resistant strain ATCC 25922 became highly susceptible to 4′sT, exhibiting a similar MIC as the susceptible strain MG1655 (Fig. 6a and Table 3). The SOS response was also induced in ATCC 25922 by 4′sT under these conditions (Fig. S8). Similarly, under sublethal TMP treatment (0.25× MIC) without thymidine supplementation, both ATCC 25922 and MG1655 strains were highly susceptible and showed similar MICs for 4′sT (Fig. 6b and Table 3). The uropathogenic isolate *E. coli* ATCC 700928 and the fecal isolate *E. coli* ATCC 43888, which are resistant to 4′sT, also became susceptible to 4′sT under TMP treatment (Table 3). To determine whether this effect extends beyond *E. coli*, we tested additional species. Previously resistant Gram-negative strains, *S. maltophilia* ATCC 13637 and *K. pneumoniae* ATCC 700603, as well as the Gram-positive *S. aureus* HG003, also became highly susceptible to 4′sT under TMP treatment. In contrast, *A. baumannii,* which lacks the thymidine salvage pathway, remained resistant under the same conditions. As a result, these findings indicate that selectivity of 4′sT is determined by the presence of the thymidine salvage pathway and the metabolic state of *de novo* thymidylate synthesis.

**TABLE 3:** MICs of 4′sT under conditions of impaired *de novo* thymidylate synthesis by TMP^*a*^

Strain	4′sT (µg/mL)	Trimethoprim (µg/mL)	4′sT (µg/mL)	
			+ Trimethoprim 4× MIC + Thymidine 12.5 µg/mL	+ Trimethoprim 0.25× MIC
*Escherichia coli* MG1655	0.0625	0.25	1	0.0156
*Escherichia coli* ATCC 25922	>128	1	0.5	0.0313
*Escherichia coli* ATCC 700928	>128	2	1	0.0313
*Escherichia coli* ATCC 43888	>128	0.125	2	0.0078
*Stenotrophomonas maltophilia* ATCC 51331	2	8	n.d.*^b^*	0.5
*Stenotrophomonas maltophilia* ATCC 13637	>128	16	n.d.*^b^*	0.125
*Klebsiella pneumoniae* ATCC 700603	>128	2	2	0.0625
*Staphylococcus aureus* HG003	>128	1	0.25	0.0039
*Acinetobacter baumannii* ATCC 17978	>128	4	n.d.*^b^*	>128

^*a*^ MICs of 4′sT were determined in the presence of TMP to inhibit the *de novo* thymidylate pathway. TMP was applied at 4× MIC in the presence of exogenous thymidine (12.5 µg/mL) or at 0.25× MIC without thymidine supplementation. n.d., not determined.

^*b*^ Thymidine supplementation did not rescue growth.

## DISCUSSION

Most nucleoside analogs developed to date are synthetic compounds, primarily used as antiviral agents. The notable example is AZT, an FDA-approved HIV drug, that contains an azido group (N₃) replacing the hydroxyl group at the 3′-position of the sugar, thereby terminating DNA replication (61). Naturally occurring nucleoside analogs, predominantly from *Streptomyces*, exhibit diverse structures and bioactivity. Representative examples include pentostatin, an FDA-approved anticancer adenosine analog, showdomycin, a C-glycosylated uridine analog inhibiting RNA synthesis, and muraymycin, a uridine-peptide hybrid targeting cell wall biosynthesis (62, 63). Here, we identify 4′sT, a sulfur-containing thymidine analog, from *Photorhabdus* with strain-selective antibacterial activity. Naturally occurring 4′-thionucleoside compounds are rare, with albomycin, a 4′-thiocytidine-containing antibiotic, being the only known example. Recent studies have elucidated the biosynthetic mechanism of the thiofuranose core in albomycin, in which the radical SAM enzyme AbmM catalyzes a sulfur-for-oxygen swapping reaction using its [Fe₄S₄] cluster as the sulfur donor (64, 65). Interestingly, the radical SAM enzymes encoded in the BGCs of 4′sT and albomycin do not share significant homology, despite both compounds featuring a 4′-sulfur modification. Genome mining revealed that similar BGCs of 4′sT are present in a range of bacterial taxa, particularly within the Enterobacterales. These uncharacterized clusters represent an interesting subject for future investigation.

4′sT exhibits strong strain-selective activity. For many nucleoside analogs, species-selective antibacterial activity is common, and the spectrum is determined by the presence of salvage pathway enzymes or their substrate preferences (66). For example, we previously reported that the guanosine analog 3′-amino 3′-deoxyguanosine that incorporates into RNA and terminates chain elongation requires guanosine kinase (*gsk*) for activation, and its spectrum of activity corresponds with species that encode this enzyme (67). In the case of 4′sT, however, the strain-selective activity was puzzling because thymidine kinase (*tdk*), which is required for 4′sT activation, is broadly conserved among *E. coli* strains, including those that are resistant. We found that impairment of *de novo* thymidylate synthesis by TMP treatment rendered these resistant strains highly susceptible to 4′sT. This observation suggests the possibility that resistant strains, including *E. coli* clinical isolates, may possess more robust *de novo* thymidylate synthesis, thereby diminishing their reliance on the salvage pathway and limiting the activation of 4′sT. Whether TMP treatment enhances the salvage pathway—for example, by upregulating *tdk* expression—remains to be determined. Nevertheless, inhibition of the *de novo* pathway is expected to alter nucleotide pool dynamics, increasing the relative abundance of 4′sT-TP relative to intracellular dTTP, thereby promoting its incorporation into DNA.

4′-Thionucleosides or 4′-thionucleotides have been synthesized to expand the utility of nucleic acids in various biological systems, including applications in gene regulation, therapeutics, and synthetic biology (68). Neither naturally occurring 4′sT nor its antibacterial activity has been previously described, but its radiolabeled synthetic forms have been studied as a PET tracer to monitor DNA synthesis in tumor models (69). It was selected for this application due to its enhanced pharmacokinetic and metabolic stability compared to traditional chain-terminating analogs. These same properties may also contribute to its potential utility as an antimicrobial compound, although *in vivo* efficacy has not yet been evaluated. In the previous studies on 4′-thionucleotides, *in vitro* PCR assays showed that 4′sT-TP is incorporated efficiently by DNA polymerases lacking 3′→5′ proofreading exonuclease activity, indicating that 4′sT-TP itself is not intrinsically chain-terminating (70). Structural analysis revealed that fully modified 4′-thioDNAs introduce steric bulk and electronic shift that cause changes in helix conformation (71). Notably, our cell-based experiments showed that 4′sT rapidly inhibits DNA replication and induces DNA damage response in *E. coli*. This suggests that incorporation of 4′sT-TP might distort the DNA or its interaction with the polymerase under cellular conditions, potentially causing transient pausing and repair responses that are not evident in bulk PCR assays. Further investigation is needed to elucidate the precise mechanism by which 4′sT inhibits DNA replication in bacteria.

The discovery of 4′sT required overcoming several challenges that are commonly encountered in systematic antibiotic discovery from natural products, such as loss of activity during subsequent cultures and dereplication of known compounds. To address these issues, we applied the EMS-Rif approach using a resistant *E. coli* mutant strain, which enabled both activation of compound production and efficient screening for EMS-Rif mutants that produce the target compound. From our experience, particularly when dealing with an unknown active compound produced at a low level, isolating mutants resistant to an extract is often more feasible than purifying the compound because it does not require a high-purity compound. Once obtained, such resistant mutants can then be used in targeted screening within the EMS-Rif workflow. We propose that the EMS-Rif strategy can also be broadly applied not only to activate the production of target compounds, but also to discover novel bioactive metabolites.

These findings underscore the continued value of exploring underexploited microbial taxa using systematic screening platforms for the discovery of new antibiotics active against Gram-negative bacteria.

## MATERIALS AND METHODS

### Screening conditions

Screening of *Photorhabdus* and *Xenorhabdus* strains was performed as previously described (67). Briefly, strains were cultured overnight at 28°C in 10 mL of LB broth with shaking at 200 rpm. Cultures were then diluted 1:100 into fresh LB or tryptic soy broth (TSB) and incubated for 8 days at 28°C with shaking at 200 rpm. After incubation, cultures were centrifuged at 8,000 × *g* for 15 min to remove cells, and the resulting supernatants were concentrated 20-fold using a Genevac evaporator and resuspended in water. Concentrated extracts were subjected to reverse-phase HPLC (Agilent 1260 HPLC system, Agilent Technologies) using a C18 semipreparative column (XBridge BEH C18, 5 µm, 10 × 250 mm). Separation was achieved using a linear gradient of acetonitrile in water, both containing 0.1% formic acid, with the following profile: 2% acetonitrile for 0–5 min, followed by a gradient from 2% to 50% acetonitrile over 5–38 min, at a flow rate of 4 mL/min. Fractions were collected at 1 min intervals, dried, and resuspended in 50 µL of water. For bioactivity assays, *E. coli* MG1655 and *S. aureus* HG003 were each grown to exponential phase in cation-adjusted Mueller-Hinton II broth (MHIIB), adjusted to an OD₆₀₀ of 0.03, and uniformly spread onto separate MHII agar plates. After the surface had dried, 6 µL of each resuspended HPLC fraction was spotted onto the lawn. Plates were incubated at 37°C for 16–20 h, and zones of inhibition were assessed visually.

### EMS-Rif approach

Overnight cultures of *P. asymbiotica* KLE11370 (GenBank genome accession number: JBREXM000000000) were prepared by inoculating a single colony into LB broth and incubating at 28°C with shaking at 200 rpm. These cultures were then diluted 1:100 into fresh LB and grown under the same conditions until an OD₆₀₀ of approximately 0.8 was reached. Cultures were subsequently chilled on ice for 30 min, centrifuged at 6,000 × *g* for 20 min at 4°C, and washed twice with 10 mL of Buffer A (10.5 g/L K₂HPO₄, 4.5 g/L KH₂PO₄, 1 g/L (NH₄)₂SO₄, and 0.5 g/L sodium citrate dihydrate, pH 7.3). The resulting cell pellets were resuspended in 10 mL of Buffer A supplemented with 1.4% (vol/vol) EMS in 15 mL screw-cap tubes and incubated at 28°C with shaking at 220 rpm for either 60 or 120 min. Following mutagenesis, the cells were pelleted again by centrifugation, washed twice with Buffer A, and resuspended in 0.5 mL of the same buffer. Aliquots (50 or 450 µL) were inoculated into 5 mL of LB and incubated overnight at 28°C for recovery. Recovered cultures were adjusted to OD₆₀₀ ~0.8, and 1 mL of each culture was spread onto LB agar plates supplemented with 50, 100, or 200 µg/mL rifampicin. Plates were incubated at 28°C for 2 days to allow selection of rifampicin-resistant colonies, which were subsequently isolated for downstream bioactivity screening. A total of 768 resistant mutants were inoculated into 2 mL deep-well 96-well plates containing 400 µL of TSB per well and cultured for 8 days at 28°C with shaking at 220 rpm. After incubation, plates were centrifuged at 8,000 × *g* for 10 min, and the resulting supernatants were dried and resuspended in 50 µL of water. To evaluate antibacterial activity, 3 µL of each resuspended sample was spotted onto MHII agar plates seeded with *E. coli* MG1655 and the MG1655-derived mutant resistant to the original active fraction. Supernatants that exhibited selective or stronger inhibitory activity against wild-type MG1655, compared to the resistant strain, were considered potential hits. Mutant-12 was regrown, and its supernatant was fractionated by reverse-phase HPLC under the same conditions described in the initial screening. The resulting fractions were tested for bioactivity by spotting 3 µL onto MHII agar plates seeded with *E. coli* MG1655 and the resistant mutant.

### Purification and identification of 4′-thiothymidine (4′sT)

A single colony of *P. asymbiotica* mutant-12 was inoculated into 10 mL of TSB and incubated overnight at 28°C with shaking at 220 rpm. The resulting preculture (10 mL) was transferred into 1 L of TSB in a 2 L Erlenmeyer flask. A total of 16 flasks (16 L in total) were incubated under the same conditions for 8 days. After incubation, cells were removed by centrifugation at 8,000 × *g* for 10 min, and the culture supernatant was directly subjected to ion-exchange chromatography on SP Sepharose XL (GE Healthcare). The active compound was eluted using 50 mM ammonium acetate buffer (pH 5), and the active fraction was further purified by reversed-phase HPLC (XBridge BEH C18, 5 µm, 19 × 250 mm) using the following conditions: solvent A, Milli-Q water with 0.1% formic acid; solvent B, acetonitrile with 0.1% formic acid. UV detection was performed at 210 and 280 nm. The gradient started with 2% solvent B for 5 min, followed by a linear increase to 60% over 20 min at a flow rate of 8 mL/min, with the active compound eluting at 16 min. This fraction was further purified (XBridge BEH C18, 5 µm, 10 × 250 mm) using a gradient from 2% to 35% solvent B over 13 min, following an initial isocratic hold at 2% for 3 min, at a flow rate of 4 mL/min, yielding an active peak at 11 min. A third HPLC purification (XBridge BEH C18, 5 µm, 10 × 250 mm) was carried out with a linear gradient from 2% to 27% over 17 min, following a 3 min isocratic phase at 2%, at a flow rate of 4 mL/min, resulting in a peak at 13 min. The final purification was performed on a Cosmosil Cholester column (5 µm, 10 × 250 mm) using a linear gradient from 4% to 25% solvent B over 27 min at 4 mL/min, and the final product, 4′sT, was eluted at 15.5 min with a final yield of ~0.1 mg/L. The ¹H and ¹H–¹H COSY NMR spectra were recorded on a Bruker Avance Neo 500 MHz NMR spectrometer equipped with a broadband BBFO probe. The experiment was performed using 0.25 mg of purified 4′sT dissolved in 600 µL of D₂O. Chemical shifts were referenced to the residual solvent peak (δH 4.8). The NMR spectra of synthetic 4′sT (Wuxi AppTec) exhibited identical chemical shifts, splitting patterns, and coupling constants to those of the purified compound. LC-MS analysis was conducted on an Agilent HPLC 1260 Infinity II coupled to an Agilent 6530 quadrupole time-of-flight mass spectrometer with electrospray ionization. An Agilent Poroshell 120 EC-C18 (2.1 × 150 mm, 2.7 µm) was utilized for the separation with the mobile phases of (A) water + 0.1% formic acid (vol/vol) and (B) acetonitrile + 0.1% formic acid (vol/vol) at a flow rate of 0.2 mL/min. Initial concentration of 2% solvent B was kept for 3 min, followed by a linear gradient to 50% for 20 min. MS parameters were as follows: collision gas, N_2_; gas temperature, 300°C; gas flow, 7 L min^−1^; nebulizer, 35 psi; fragmentor voltage, 175 V; skimmer voltage, 65 V. Acquisition was set in positive mode at “Auto MS/MS” with the following parameters: mass range for MS 111–3,000 m/z with two spectra per second; mass range for MSMS, 50–3,000 m/z with four spectra per second; collision energy, “use formula” mode, slope = 1 and offset = 10; max precursor per cycle, 10; active exclusion after three spectra with released after 0.5 min. Data acquisition and analysis were performed using Agilent MassHunter Data Acquisition Software version 10.1 and Agilent MassHunter Qualitative Analysis Software version 10.0, respectively.

### Antibiotic susceptibility testing

Susceptibility testing for aerobic and facultative anaerobic bacteria was performed using MHIIB. Overnight cultures were diluted 1:100 into fresh MHIIB and incubated at 37°C with shaking until reaching mid-exponential phase. Cultures were then adjusted to an OD₆₀₀ of 0.0001, and 100 µL was dispensed into 96-well microtiter plates containing serial twofold dilutions of test compounds. Plates were incubated at 37°C for 16 to 20 h under aerobic conditions. Growth was assessed both visually and by measuring OD₆₀₀. The MIC was defined as the lowest concentration of compound at which no visible growth and no increase in OD₆₀₀ were observed. For anaerobic gut bacteria, strains were revived from frozen stocks in Hungate tubes containing Gifu anaerobic medium (GAM) supplemented with 0.5 g/L L-cysteine and 0.0001% resazurin, and incubated anaerobically at 37°C for 3 days. Cultures were then passaged 1:100 into fresh medium and incubated for 24 h prior to MIC testing. On the day of the assay, cultures were diluted 1:1,000 into fresh GAM supplemented as above and transferred to 96-well plates containing serial dilutions of test compounds. Plates were sealed and incubated anaerobically at 37°C for 24 h. Growth was assessed visually.

### Cytotoxicity testing

Cytotoxicity was evaluated using the Alamar Blue assay in 96-well plates. Primary human lung fibroblasts and mammalian cancer cell lines (HepG2, FaDu, HEK293, and A549) were obtained from the American Type Culture Collection (ATCC) and cultured according to the manufacturer’s recommended media and conditions. Cells were seeded in their respective growth media and allowed to adhere for 24 h at 37°C in a humidified incubator with 5% CO₂. 4′sT was then added in a twofold serial dilution. A 1:1 (vol/vol) mixture of DMSO and growth medium was included as a positive control for cytotoxicity, while untreated cells maintained in complete growth medium served as the negative (viability) control. Plates were incubated for 72 h under the same conditions. Following incubation, 3 mM resazurin was added to each well and incubated for an additional 3 h. Absorbance was measured at 544 and 590 nm using a microplate reader to assess cell viability (BioTek).

### Heterologous expression of 4′sT

The expression system was cloned into an in-house domesticated pRSFDuet-1 (Merck). pRSFDuet-1 was selected for its ability to handle large BGCs. *P*. *asymbiotica* DSM15149 (GenBank accession number GCF_000196475.1) chromosomal DNA was used as a template in all steps; it was extracted using the DNeasy Blood & Tissue Kit (Qiagen). In general, fragments were amplified using Q5 DNA polymerase (New England Biolabs) and purified from the agarose gel using the Zymoclean Gel DNA Recovery Kit (Zymo Research). The polymerase chain reaction (PCR) was performed in an Eppendorf Mastercycler (Eppendorf) using the following program: 95°C for 2 min; 35 cycles of 95°C for 10 s, 67°C for 30 s, 72°C for 1 min/kb (extension time varied depending on the length of the fragment to be amplified), followed by a final extension step at 72°C for 10 min. All primer sequences can be found in Table S2. The first expression vector pRSF*-thmA-C* was constructed using a domesticated pRSFDuet-1 linearized at the PacI restriction enzyme site (New England Biolabs) and purified from an agarose gel using the Zymoclean Gel DNA Recovery Kit. The *thmA-C* gene fragment was amplified using primers F1 and R1 (Table S2). The gel-purified fragment was cloned into the first multiple cloning site under the control of the T7-lac promoter. This was achieved by Gibson assembly (New England Biolabs) with a 3:1 molar ratio of insert to linearized vector backbone at 50°C for 30 min. After assembly, the reaction was placed on ice and transformed into chemically competent *E. coli* DH5α (New England Biolabs) as the plasmid maintenance host. The transformants were selected on kanamycin (50 µg/mL) LBA plates. Several single colonies were used to inoculate LBB with selective pressure for plasmid extraction; this was performed using a 4 mL overnight culture and the Monarch DNA Purification Kit (New England Biolabs). The plasmids were sent for sequencing (Primordium). The plasmids were verified by sequence alignment, and subsequently, pRSF*-thmA-C* was transformed into the expression system, *E. coli* BL21(DE3) (New England Biolabs). 4′sT was expressed in formulated media (12.54 g/L K₂HPO₄, 2.31 g/L KH₂PO₄, 5 g/L NaCl, 12 g/L yeast extract, 4 g/L D(+) glucose, 1 g/L NH₄Cl, 0.24 g/L MgSO₄, 1 mg/L sterile-filtered vitamin B₁₂, pH 7.0). Overnight seeds were generated as 10 mL LBB with selective pressure, which was back-diluted into 1 L fermentations in baffled wide-neck flasks at 30°C, 150 rpm for 3 days. At the point of inoculation in the fermenter, IPTG was added at a final concentration of 0.1 mM. In the same manner, to confirm *thmC* as the self-resistance gene, pRSF-*thmC* was cloned, but the *thmC* fragment was amplified using F2 and R2 (Table S2).

### Time-kill assays

An overnight culture of *E. coli* MG1655 in MHIIB was diluted 1:10,000 into fresh medium and incubated at 37°C with shaking at 220 rpm for 2 h. Cultures were then treated with 16× MIC of 4′sT. At designated time points, 100 µL aliquots were collected, centrifuged at 8,000 × *g* for 3 min, washed with 100 µL phosphate-buffered saline (PBS), and resuspended in 100 µL PBS. Tenfold serial dilutions were plated on MHII agar, and colonies were enumerated after overnight incubation at 37°C to determine CFU/mL. Experiments were performed in biological triplicate.

### Resistance studies

Exponential-phase cultures of *E. coli* MG1655 in MHIIB were washed with PBS and plated onto MHII agar containing 64 or 16 µg/mL of 4′sT at final cell densities of 1.5 × 10⁶ and 1.5 × 10⁷ CFU per plate. After 48 h of incubation at 37°C, colonies were counted and restreaked to assess resistance stability and determine MIC values. Genome sequencing and variant calling for mutants were performed by SeqCenter (Pittsburgh, PA). Library preparation was conducted using the Illumina DNA Prep Kit with IDT 10 bp unique dual indices. Libraries were sequenced on the Illumina NextSeq 6000 platform to generate 2 × 150 bp paired-end reads. Reads were aligned to the *E. coli* MG1655 reference genome, and variants were identified using the breseq software package.

### LC-MS analysis of incorporated 4′sT

*E. coli* MG1655 was grown overnight in MHIIB at 37°C with shaking at 220 rpm, then diluted 1:100 into fresh MHIIB and incubated for 2 h. 4′sT (16 or 4 µg/mL) was added, and cultures were further incubated for 1 h. Cells were pelleted, and genomic DNA was purified using the DNeasy Blood & Tissue Kit (Qiagen). DNA (20 µg) was digested with NEB Nucleoside Digestion Mix overnight at 37°C, filtered through a 0.22 µm membrane (Millipore), and analyzed by LC-MS/MS to quantify 4′sT incorporation. The same procedure was performed for *E. coli* ATCC 25922.

### EdU incorporation assay

Overnight cultures of *E. coli* MG1655 or ATCC 25922 were diluted 1:100 into fresh MHIIB and incubated at 37°C with shaking at 220 rpm for 2 h (OD_600_ = 0.3–0.4). Cultures were then treated with 4′sT (0.25 µg/mL), thymidine (0.25 µg/mL), or ciprofloxacin (2 µg/mL). At 5 and 25 min post-treatment, 500 µL aliquots were harvested and immediately added to 15 mL tubes containing 5-ethynyl-2′-deoxyuridine (EdU) at a final concentration of 40 µg/mL. After a 5 min incubation with EdU at 37°C, cells were fixed with 4% paraformaldehyde for 20 min at room temperature. Following fixation, cells were pelleted by centrifugation, washed twice with PBS, and subsequently permeabilized by incubation in 0.5% Triton X-100 in PBS for 20 min at room temperature. Cells were then washed again with PBS to remove residual detergent. EdU incorporation was detected using the Click-iT EdU Alexa Fluor 488 Flow Cytometry Assay Kit (Thermo Fisher Scientific) according to the manufacturer’s protocol. Briefly, cells were incubated with the click reaction cocktail for 30 min in the dark at room temperature. After washing, cells were resuspended in PBS and analyzed by FACSAria II flow cytometer (BD Biosciences). Data were processed using FlowJo v10 software.

### Supercoiling assay

DNA supercoiling was assessed using the fluorescence-based reporter plasmid pSupR, as previously described (55). Briefly, *E. coli* MG1655 carrying pSupR was grown overnight in LB supplemented with 50 µg/mL ampicillin, diluted 1:100 into fresh LB, and incubated at 37°C with shaking at 220 rpm until mid-exponential phase. Cultures were then treated with 1× or 2× MIC of 4′sT and further incubated. GFP fluorescence was measured using a plate reader (BioTek) and normalized to OD_600_.

### SOS response assay

*E. coli* MG1655 and ATCC 25922 carrying the pDualrep2 plasmid were grown overnight, diluted 1:100 into MHIIB supplemented with 50 µg/mL ampicillin, and incubated at 37°C for 2 h with shaking at 220 rpm. Cultures were then diluted to an OD_600_ of 0.001 in fresh MHIIB and transferred to 96-well black clear-bottom plates. 4′sT was added to the wells at the indicated concentrations. Red fluorescent protein (RFP) expression, driven by the *sulA* promoter, and OD₆₀₀ were measured every 15 min for 960 min using a Synergy H1 microplate reader (BioTek). For agar-based assays, *E. coli* MG1655 and ATCC 25922 harboring pDualrep2 were grown to the exponential phase, adjusted to an OD₆₀₀ of 0.03, and spread evenly onto MHII agar plates. After the surface dried, 4′sT was spotted onto the lawn. Plates were incubated for 16 h at 37°C, and RFP fluorescence was measured using the Cy3 channel on a ChemiDoc imaging system (Bio-Rad).

## SUPPLEMENTAL MATERIAL

Supplemental Material mbio.02164-25-s0001.pdf Supplemental figures and tables.

ASM does not own the copyrights to Supplemental Material that may be linked to, or accessed through, an article. The authors have granted ASM a non-exclusive, world-wide license to publish the Supplemental Material files. Please contact the corresponding author directly for reuse.

## References

[1] Antimicrobial Resistance Collaborators. 2022. Global burden of bacterial antimicrobial resistance in 2019: a systematic analysis. Lancet. doi:10.1016/S0140-6736(21)02724-0 37500506

[2] Sati H, Carrara E, Savoldi A, Hansen P, Garlasco J, Campagnaro E, Boccia S, Castillo-Polo JA, Magrini E, Garcia-Vello P, Wool E, Gigante V, Duffy E, et al.. 2025. The WHO bacterial priority pathogens list 2024: a prioritisation study to guide research, development, and public health strategies against antimicrobial resistance. Lancet Infect Dis. doi:10.1016/S1473-3099(25)00118-5 PMC12367593 40245910

[3] Zgurskaya HI, Rybenkov VV, Krishnamoorthy G, Leus IV. 2018. Trans-envelope multidrug efflux pumps of gram-negative bacteria and their synergism with the outer membrane barrier. Res Microbiol 169:351–356. doi:10.1016/j.resmic.2018.02.002 29454787 PMC6095828

[4] Gould K. 2016. Antibiotics: from prehistory to the present day. J Antimicrob Chemother 71:572–575. doi:10.1093/jac/dkv484 26851273

[5] Li JW-H, Vederas JC. 2009. Drug discovery and natural products: end of an era or an endless frontier? Science 325:161–165. doi:10.1126/science.1168243 19589993

[6] Jones MB, Nierman WC, Shan Y, Frank BC, Spoering A, Ling L, Peoples A, Zullo A, Lewis K, Nelson KE. 2017. Reducing the bottleneck in discovery of novel antibiotics. Microb Ecol 73:658–667. doi:10.1007/s00248-016-0889-3 27896376

[7] Crits-Christoph A, Diamond S, Butterfield CN, Thomas BC, Banfield JF. 2018. Novel soil bacteria possess diverse genes for secondary metabolite biosynthesis. Nature 558:440–444. doi:10.1038/s41586-018-0207-y 29899444

[8] Kautsar SA, van der Hooft JJJ, de Ridder D, Medema MH. 2021. BiG-SLiCE: a highly scalable tool maps the diversity of 1.2 million biosynthetic gene clusters. Gigascience 10:giaa154. doi:10.1093/gigascience/giaa154 33438731 PMC7804863

[9] Sajnaga E, Kazimierczak W, Karaś MA, Jach ME. 2024. Exploring *Xenorhabdus* and *Photorhabdus* nematode symbionts in search of novel therapeutics. Molecules 29:5151. doi:10.3390/molecules29215151 39519791 PMC11547657

[10] Meesil W, Muangpat P, Sitthisak S, Rattanarojpong T, Chantratita N, Machado RAR, Shi YM, Bode HB, Vitta A, Thanwisai A. 2023. Genome mining reveals novel biosynthetic gene clusters in entomopathogenic bacteria. Sci Rep 13:20764. doi:10.1038/s41598-023-47121-9 38007490 PMC10676414

[11] Shi Y-M, Hirschmann M, Shi Y-N, Ahmed S, Abebew D, Tobias NJ, Grün P, Crames JJ, Pöschel L, Kuttenlochner W, Richter C, Herrmann J, et al.. 2022. Global analysis of biosynthetic gene clusters reveals conserved and unique natural products in entomopathogenic nematode-symbiotic bacteria. Nat Chem 14:701–712. doi:10.1038/s41557-022-00923-2 35469007 PMC9177418

[12] Imai Y, Meyer KJ, Iinishi A, Favre-Godal Q, Green R, Manuse S, Caboni M, Mori M, Niles S, Ghiglieri M, et al.. 2019. A new antibiotic selectively kills gram-negative pathogens. Nature 576:459–464. doi:10.1038/s41586-019-1791-1 31747680 PMC7188312

[13] Pantel L, Florin T, Dobosz-Bartoszek M, Racine E, Sarciaux M, Serri M, Houard J, Campagne J-M, de Figueiredo RM, Midrier C, Gaudriault S, Givaudan A. 2018. Odilorhabdins, antibacterial agents that cause miscoding by binding at a new ribosomal site. Mol Cell 70:83–94. doi:10.1016/j.molcel.2018.03.001 29625040

[14] Imai Y, Hauk G, Quigley J, Liang L, Son S, Ghiglieri M, Gates MF, Morrissette M, Shahsavari N, Niles S, Baldisseri D, Honrao C, Ma X, et al.. 2022. Evybactin is a DNA gyrase inhibitor that selectively kills *Mycobacterium tuberculosis*. Nat Chem Biol 18:1236–1244. doi:10.1038/s41589-022-01102-7 35996001 PMC9844538

[15] Liang L, Quigley J, Theriault M, Iinishi A, Bargabos R, Morrissette M, Ghiglieri M, Curtis T, Corsetti R, Son S, Sarkar B, Lewis K. 2025. A chlorinated diketopiperazine antibiotic targets *Mycobacterium tuberculosis* DNA Gyrase. bioRxiv:2025.03.10.642354. doi:10.1101/2025.03.10.642354 PMC12406682 40741954

[16] Bargabos R, Iinishi A, Hawkins B, Privalsky T, Pitt N, Son S, Corsetti R, Gates MF, Miller RD, Lewis K. 2024. Small molecule produced by *Photorhabdus* interferes with ubiquinone biosynthesis in gram-negative bacteria. mBio 15:e0116724. doi:10.1128/mbio.01167-24 39254306 PMC11481567

[17] Cardona ST, Rahman ASMZ, Novomisky Nechcoff J. 2025. Innovative perspectives on the discovery of small molecule antibiotics. NPJ Antimicrob Resist 3:19. doi:10.1038/s44259-025-00089-0 40082593 PMC11906701

[18] World Health Organization. 2024. 2023 Antibacterial agents in clinical and preclinical development: an overview and analysis. World Health Organization. https://www.who.int/publications/i/item/9789240094000.

[19] Lewis K. 2020. The science of antibiotic discovery. Cell 181:29–45. doi:10.1016/j.cell.2020.02.056 32197064

[20] Willing BP, Russell SL, Finlay BB. 2011. Shifting the balance: antibiotic effects on host-microbiota mutualism. Nat Rev Microbiol 9:233–243. doi:10.1038/nrmicro2536 21358670

[21] Diamantis S, Retur N, Bertrand B, Lieutier-Colas F, Carenco P, Mondain V, Promise Professional Community Network On Antimicrobial R. 2022. The production of antibiotics must be reoriented: repositioning old narrow-spectrum antibiotics. Antibiotics (Basel) 11. doi:10.3390/antibiotics11070924 PMC9311687 35884178

[22] Chain C, Sheehan JP, Xu X, Ghaffari S, Godbole A, Kim H, Freundlich JS, Rabinowitz JD, Gitai Z. 2024. A folate inhibitor exploits metabolic differences in *Pseudomonas aeruginosa* for narrow-spectrum targeting. Nat Microbiol 9:1207–1219. doi:10.1038/s41564-024-01665-2 38594311 PMC11087268

[23] Zampaloni C, Mattei P, Bleicher K, Winther L, Thäte C, Bucher C, Adam J-M, Alanine A, Amrein KE, Baidin V, et al.. 2024. A novel antibiotic class targeting the lipopolysaccharide transporter. Nature 625:566–571. doi:10.1038/s41586-023-06873-0 38172634 PMC10794144

[24] Kan J, Morales-Amador A, Hernandez Y, Ternei MA, Lemetre C, Maclntyre LW, Biais N, Brady SF. 2025. Oxydifficidin, a potent *Neisseria gonorrhoeae* antibiotic due to DedA-assisted uptake and ribosomal protein RplL sensitivity. elife 13:RP99281. doi:10.7554/eLife.99281 40433956 PMC12119084

[25] Wittke F, Vincent C, Chen J, Heller B, Kabler H, Overcash JS, Leylavergne F, Dieppois G. 2020. Afabicin, a first-in-class antistaphylococcal antibiotic, in the treatment of acute bacterial skin and skin structure infections: clinical noninferiority to vancomycin/linezolid. Antimicrob Agents Chemother 64:e00250-20. doi:10.1128/AAC.00250-20 32747361 PMC7508579

[26] Martin-Loeches I, Dale GE, Torres A. 2018. Murepavadin: a new antibiotic class in the pipeline. Expert Rev Anti Infect Ther 16:259–268. doi:10.1080/14787210.2018.1441024 29451043

[27] Guo L, Wambui J, Wang C, Broos J, Stephan R, Kuipers OP. 2024. Rombocin, a short stable natural nisin variant, displays selective antimicrobial activity against *Listeria monocytogenes* and employs a dual mode of action to kill target bacterial strains. ACS Synth Biol 13:370–383. doi:10.1021/acssynbio.3c00612 38194633 PMC10804407

[28] Muñoz KA, Ulrich RJ, Vasan AK, Sinclair M, Wen P-C, Holmes JR, Lee HY, Hung C-C, Fields CJ, Tajkhorshid E, Lau GW, Hergenrother PJ. 2024. A gram-negative-selective antibiotic that spares the gut microbiome. Nature 630:429–436. doi:10.1038/s41586-024-07502-0 38811738 PMC12135874

[29] Leimer N, Wu X, Imai Y, Morrissette M, Pitt N, Favre-Godal Q, Iinishi A, Jain S, Caboni M, Leus IV, et al.. 2021. A selective antibiotic for lyme disease. Cell 184:5405–5418. doi:10.1016/j.cell.2021.09.011 34619078 PMC8526400

[30] Chioti VT, McWhorter KL, Blue TC, Li Y, Xu F, Jeffrey PD, Davis KM, Seyedsayamdost MR. 2024. Potent and specific antibiotic combination therapy against *Clostridioides difficile*. Nat Chem Biol 20:924–933. doi:10.1038/s41589-024-01651-z 38942968 PMC11306116

[31] Jangra M, Travin DY, Aleksandrova EV, Kaur M, Darwish L, Koteva K, Klepacki D, Wang W, Tiffany M, Sokaribo A, Chen X, Deng Z, Tao M, et al.. 2025. A broad-spectrum lasso peptide antibiotic targeting the bacterial ribosome. Nature 640:1022–1030. doi:10.1038/s41586-025-08723-7 40140562 PMC12497486

[32] Mao D, Okada BK, Wu Y, Xu F, Seyedsayamdost MR. 2018. Recent advances in activating silent biosynthetic gene clusters in bacteria. Curr Opin Microbiol 45:156–163. doi:10.1016/j.mib.2018.05.001 29883774 PMC6281788

[33] Dinglasan JLN, Otani H, Doering DT, Udwary D, Mouncey NJ. 2025. Microbial secondary metabolites: advancements to accelerate discovery towards application. Nat Rev Microbiol 23:338–354. doi:10.1038/s41579-024-01141-y 39824928

[34] Bode HB, Bethe B, Höfs R, Zeeck A. 2002. Big effects from small changes: possible ways to explore nature’s chemical diversity. Chembiochem 3:619–627. doi:10.1002/1439-7633(20020703)3:7<619::AID-CBIC619>3.0.CO;2-9 12324995

[35] Gram L, Melchiorsen J, Bruhn JB. 2010. Antibacterial activity of marine culturable bacteria collected from a global sampling of ocean surface waters and surface swabs of marine organisms. Mar Biotechnol (NY) 12:439–451. doi:10.1007/s10126-009-9233-y 19823914

[36] Park SR, Tripathi A, Wu J, Schultz PJ, Yim I, McQuade TJ, Yu F, Arevang C-J, Mensah AY, Tamayo-Castillo G, Xi C, Sherman DH. 2016. Discovery of cahuitamycins as biofilm inhibitors derived from a convergent biosynthetic pathway. Nat Commun 7:10710. doi:10.1038/ncomms10710 26880271 PMC4757757

[37] Li JY, Sidhu RS, Ford EJ, Long DM, Hess WM, Strobel GA. 1998. The induction of taxol production in the endophytic fungus— *Periconia* sp from *Torreya* grandifolia. Journal of Industrial Microbiology and Biotechnology 20:259–264. doi:10.1038/sj.jim.2900521

[38] Danner C, Mach RL, Mach-Aigner AR. 2023. The phenomenon of strain degeneration in biotechnologically relevant fungi. Appl Microbiol Biotechnol 107:4745–4758. doi:10.1007/s00253-023-12615-z 37341752 PMC10345034

[39] Tanaka Y, Kasahara K, Hirose Y, Murakami K, Kugimiya R, Ochi K. 2013. Activation and products of the cryptic secondary metabolite biosynthetic gene clusters by rifampin resistance (rpoB) mutations in actinomycetes. J Bacteriol 195:2959–2970. doi:10.1128/JB.00147-13 23603745 PMC3697537

[40] Cai XC, Xi H, Liang L, Liu JD, Liu CH, Xue YR, Yu XY. 2017. Rifampicin-resistance mutations in the rpoB gene in *Bacillus velezensis* CC09 have pleiotropic effects. Front Microbiol 08:178. doi:10.3389/fmicb.2017.00178 PMC5303731 28243227

[41] Secrist JA III, Tiwari KN, Riordan JM, Montgomery JA. 1991. Synthesis and biological activity of 2’-deoxy-4’-thio pyrimidine nucleosides. J Med Chem 34:2361–2366. doi:10.1021/jm00112a007 1652015

[42] Yoon S-H, Ha S, Lim J, Kwon S, Chun J. 2017. A large-scale evaluation of algorithms to calculate average nucleotide identity. Antonie Van Leeuwenhoek 110:1281–1286. doi:10.1007/s10482-017-0844-4 28204908

[43] Wang M, Zhang Y, Lv L, Kong D, Niu G. 2022. Biosynthesis and chemical synthesis of albomycin nucleoside antibiotics. Antibiotics (Basel) 11:438. doi:10.3390/antibiotics11040438 35453190 PMC9032320

[44] Zheng Z, Ushimaru R, Mori T, Ruszczycky MW, Abe I, Liu H. 2025. Biosynthesis of the thiofuranose core in Albomycin requires a versatile enzyme AbmG that catalyzes net dehydration via cryptic phosphorylation. J Am Chem Soc 147:34143–34149. doi:10.1021/jacs.5c12827 40907032 PMC12857199

[45] Wang J, Woldring RP, Román-Meléndez GD, McClain AM, Alzua BR, Marsh ENG. 2014. Recent advances in radical SAM enzymology: new structures and mechanisms. ACS Chem Biol 9:1929–1938. doi:10.1021/cb5004674 25009947 PMC4168785

[46] Gilchrist CLM, Booth TJ, van Wersch B, van Grieken L, Medema MH, Chooi Y-H. 2021. Cblaster: a remote search tool for rapid identification and visualization of homologous gene clusters. Bioinform Adv 1:vbab016. doi:10.1093/bioadv/vbab016 36700093 PMC9710679

[47] O’Rourke A, Beyhan S, Choi Y, Morales P, Chan AP, Espinoza JL, Dupont CL, Meyer KJ, Spoering A, Lewis K, Nierman WC, Nelson KE. 2020. Mechanism-of-action classification of antibiotics by global transcriptome profiling. Antimicrob Agents Chemother 64. doi:10.1128/AAC.01207-19 PMC7038283 31907190

[48] McKeown M, Kahn M, Hanawalt P. 1976. Thymidine uptake and utilization in *Escherichia coli*: a new gene controlling nucleoside transport. J Bacteriol 126:814–822. doi:10.1128/jb.126.2.814-822.1976 770455 PMC233218

[49] Baba T, Ara T, Hasegawa M, Takai Y, Okumura Y, Baba M, Datsenko KA, Tomita M, Wanner BL, Mori H. 2006. Construction of *Escherichia coli* K‐12 in‐frame, single‐gene knockout mutants: the Keio collection . Mol Syst Biol 2:0008. doi:10.1038/msb4100050 PMC1681482 16738554

[50] Lu Q, Zhang X, Almaula N, Mathews CK, Inouye M. 1995. The gene for nucleoside diphosphate kinase functions as a mutator gene in *Escherichia coli*. J Mol Biol 254:337–341. doi:10.1006/jmbi.1995.0620 7490752

[51] Russo TA, Umland TC, Deng X, El Mazouni F, Kokkonda S, Olson R, Carlino-MacDonald U, Beanan J, Alvarado CL, Tomchick DR, Hutson A, Chen H, Posner B, Rathod PK, Charman SA, Phillips MA. 2022. Repurposed dihydroorotate dehydrogenase inhibitors with efficacy against drug-resistant *Acinetobacter baumannii* . Proc Natl Acad Sci USA 119:e2213116119. doi:10.1073/pnas.2213116119 36512492 PMC9907071

[52] Munch-Petersen A, Mygind B, Nicolaisen A, Pihl NJ. 1979. Nucleoside transport in cells and membrane vesicles from *Escherichia coli* K12. J Biol Chem 254:3730–3737. 374403

[53] Munch-Petersen A, Mygind B. 1976. Nucleoside transport systems in *Escherichia coli* K12: specificity and regulation. J Cell Physiol 89:551–559. doi:10.1002/jcp.1040890410 827549

[54] Ashour ME, Byrum AK, Meroni A, Xia J, Singh S, Galletto R, Rosenberg SM, Vindigni A, Mosammaparast N. 2023. Rapid profiling of DNA replication dynamics using mass spectrometry-based analysis of nascent DNA. J Cell Biol 222:e202207121. doi:10.1083/jcb.202207121 36795402 PMC9960042

[55] Duprey A, Groisman EA. 2020. FEDS: a novel fluorescence-based high-throughput method for measuring DNA supercoiling *in vivo* . mBio 11:mBio doi:10.1128/mBio.01053-20 PMC7387798 32723920

[56] Osterman IA, Komarova ES, Shiryaev DI, Korniltsev IA, Khven IM, Lukyanov DA, Tashlitsky VN, Serebryakova MV, Efremenkova OV, Ivanenkov YA, Bogdanov AA, Sergiev PV, Dontsova OA. 2016. Sorting out antibiotics’ mechanisms of action: a double fluorescent protein reporter for high-throughput screening of ribosome and DNA biosynthesis inhibitors. Antimicrob Agents Chemother 60:7481–7489. doi:10.1128/AAC.02117-16 27736765 PMC5119032

[57] Crane JK, Alvarado CL, Sutton MD. 2021. Role of the SOS response in the generation of antibiotic resistance *in vivo* . Antimicrob Agents Chemother 65:e0001321. doi:10.1128/AAC.00013-21 33875437 PMC8373240

[58] Itsko M, Schaaper RM. 2011. The dgt gene of *Escherichia coli* facilitates thymine utilization in thymine-requiring strains. Mol Microbiol 81:1221–1232. doi:10.1111/j.1365-2958.2011.07756.x 21736641 PMC3195379

[59] Brogden RN, Carmine AA, Heel RC, Speight TM, Avery GS. 1982. Trimethoprim: a review of its antibacterial activity, pharmacokinetics and therapeutic use in urinary tract infections. Drugs (Abingdon Engl) 23:405–430. doi:10.2165/00003495-198223060-00001 7049657

[60] Koch AE, Burchall JJ. 1971. Reversal of the antimicrobial activity of trimethoprim by thymidine in commercially prepared media. Appl Microbiol 22:812–817. doi:10.1128/am.22.5.812-817.1971 4943586 PMC376424

[61] D’Andrea G, Brisdelli F, Bozzi A. 2008. AZT: an old drug with new perspectives. Curr Clin Pharmacol 3:20–37. doi:10.2174/157488408783329913 18690875

[62] Shiraishi T, Kuzuyama T. 2019. Recent advances in the biosynthesis of nucleoside antibiotics. J Antibiot (Tokyo) 72:913–923. doi:10.1038/s41429-019-0236-2 31554958

[63] Ren D, Ruszczycky MW, Ko Y, Wang SA, Ogasawara Y, Kim M, Liu HW. 2020. Characterization of the coformycin biosynthetic gene cluster in *Streptomyces kaniharaensis* Proc Natl Acad Sci USA 117:10265–10270. doi:10.1073/pnas.2000111117 32350138 PMC7229688

[64] Zheng Z, Ushimaru R, Thomas CM, Liu HW. 2025. Radical propagation via σ-cleavage mediates radical SAM catalyzed sulfur-for-oxygen swapping reaction during the biosynthesis of albomycin delta(2) J Am Chem Soc 147:32118–32123. doi:10.1021/jacs.5c10855 40834352 PMC12401566

[65] Ushimaru R, Zheng Z, Xiong J, Mori T, Abe I, Guo Y, Liu HW. 2025. Radical *S*-adenosyl-l-methionine FeS cluster implicated as the sulfur donor during albomycin biosynthesis. Nat Catal. doi:10.1038/s41929-025-01367-w PMC12372959 40857529

[66] Sandrini MPB, Clausen AR, On SLW, Aarestrup FM, Munch-Petersen B, Piskur J. 2007. Nucleoside analogues are activated by bacterial deoxyribonucleoside kinases in a species-specific manner. J Antimicrob Chemother 60:510–520. doi:10.1093/jac/dkm240 17615154

[67] Shahsavari N, Wang B, Imai Y, Mori M, Son S, Liang L, Böhringer N, Manuse S, Gates MF, Morrissette M, Corsetti R, Espinoza JL, Dupont CL, Laub MT, Lewis K. 2022. A silent operon of *Photorhabdus luminescens* encodes a prodrug mimic of GTP. mBio 13:e0070022. doi:10.1128/mbio.00700-22 35575547 PMC9239236

[68] Inoue N, Minakawa N, Matsuda A. 2006. Synthesis and properties of 4’-ThioDNA: unexpected RNA-like behavior of 4’-ThioDNA. Nucleic Acids Res 34:3476–3483. doi:10.1093/nar/gkl491 16855286 PMC1524900

[69] Toyohara J, Kumata K, Fukushi K, Irie T, Suzuki K. 2006. Evaluation of 4’-[methyl-14C]thiothymidine for *in vivo* DNA synthesis imaging. J Nucl Med 47:1717–1722. 17015909

[70] Kojima T, Furukawa K, Maruyama H, Inoue N, Tarashima N, Matsuda A, Minakawa N. 2013. PCR amplification of 4’-thioDNA using 2’-deoxy-4’-thionucleoside 5’-triphosphates. ACS Synth Biol 2:529–536. doi:10.1021/sb400074w 23957635

[71] Matsugami A, Ohyama T, Inada M, Inoue N, Minakawa N, Matsuda A, Katahira M. 2008. Unexpected A-form formation of 4’-thioDNA in solution, revealed by NMR, and the implications as to the mechanism of nuclease resistance. Nucleic Acids Res 36:1805–1812. doi:10.1093/nar/gkn011 18252770 PMC2330235

