# Supplementary material for: A sulfur-containing nucleoside antibiotic from Photorhabdus
Source: mBio. 2025 Oct 31;16(12):e02164-25. doi: 10.1128/mbio.02164-25 (PMC12691670; doi:10.1128/mbio.02164-25)
Supplement: Supplemental Material — Supplemental figures and tables. [file mbio.02164-25-s0001.pdf]

Supplemental material

A sulfur-containing nucleoside antibiotic from *Photorhabdus*

Sangkeun Son, Negar Shahsavari, Thomas Privalsky, Bryson A. Hawkins, Mi-Hyun Lee, Norman Pitt, Akira Iinishi, Raleb Taher, Nikita Gupta, Chandra Sekar Shankar, Jaini Patel, and Kim Lewis*

Antimicrobial Discovery Center, Department of Biology, Northeastern University, 360 Huntington Ave., Boston, MA, 02115, USA

*Corresponding Author.
E-mail: k.lewis@northeastern.edu

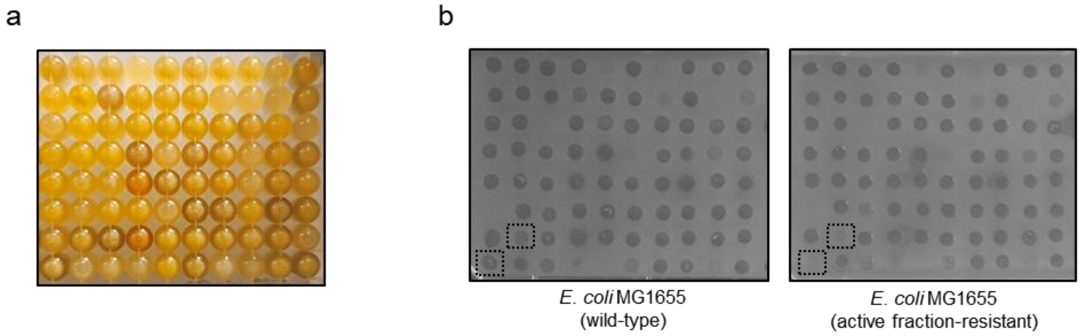

Figure S1. Representative data of EMS-Rif mutants screening.
(a) Cultures of EMS-Rif mutants derived from *P. asymbiotica* KLE11370. EMS-Rif mutants were cultured for 8 days in 400 μL of TSB medium in deep-well microplates.
(b) Bioassay of extracts from EMS-Rif mutants against *E. coli* MG1655 wild-type and active fraction-resistant strains. Hits that inhibit the wild-type strain but not the active fraction-resistant strain are indicated with dashed boxes.

Evidence	Position	Mutation	Annotation	Gene Direction	Function
RA	26,315	G→A	G336R (GGA→AGA)	astC →	Succinylornithine transaminase
RA	34,532	G→A	intergenic (+28/-8)	pspA → / → pspB	phage shock protein PspA / envelope stress response membrane protein PspB
RA	36,302	C→G	intergenic (+283/–)	hemG → / –	menaquinone-dependent protoporphyrinogen IX dehydrogenase/–
RA	65,366	G→A	S531F (TCT→TTT)	rpoB →	DNA-directed RNA polymerase subunit beta
RA	71,939	G→A	intergenic (-395/-552)	JPMAEB_11400 ← / → JPMAEB_11405	Transmembrane domain-containing protein / DUF3857 domain-containing protein
RA	81,967	G→A	A171T (GCT→ACT)	rihB →	Pyrimidine-specific ribonucleoside hydrolase RihB
RA	92,146	G→A	E119K (GAA→AAA)	JPMAEB_04075 →	GDP-mannose-dependent alpha-(1-6)-phosphatidylinositol dimannoside mannosyltransferase
RA	199,388	G→A	A614V (GCT→GTT)	JPMAEB_02235 ←	Nodulation protein

Table S1. Mutations in *Photorhabdus asymbiotica* KLE11370 Mutant-12

Primer	Sequence
F-1	GAACGCCAGCACATGGACTCGTCTACTAGCGCAGCTTAATGTCAGTCAGACGTCATGTACAAGC
R-1	TGCTAGTTATTGCTCAGCGGTGGCAGCAGCCTAGGTTAATGACTGACTGGTACCTTAAATCAGATTTCTTT
F-2	GAACGCCAGCACATGGACTCGTCTACTAGCGCAGCTTAATATGCGTCAACGCATTATTGTATGC
R-2	TGCTAGTTATTGCTCAGCGGTGGCAGCAGCCTAGGTTAATTAAATCAGATTTCTTTGCTTTAATGTTATTTTTGTTGCT

Table S2. Primer sequences

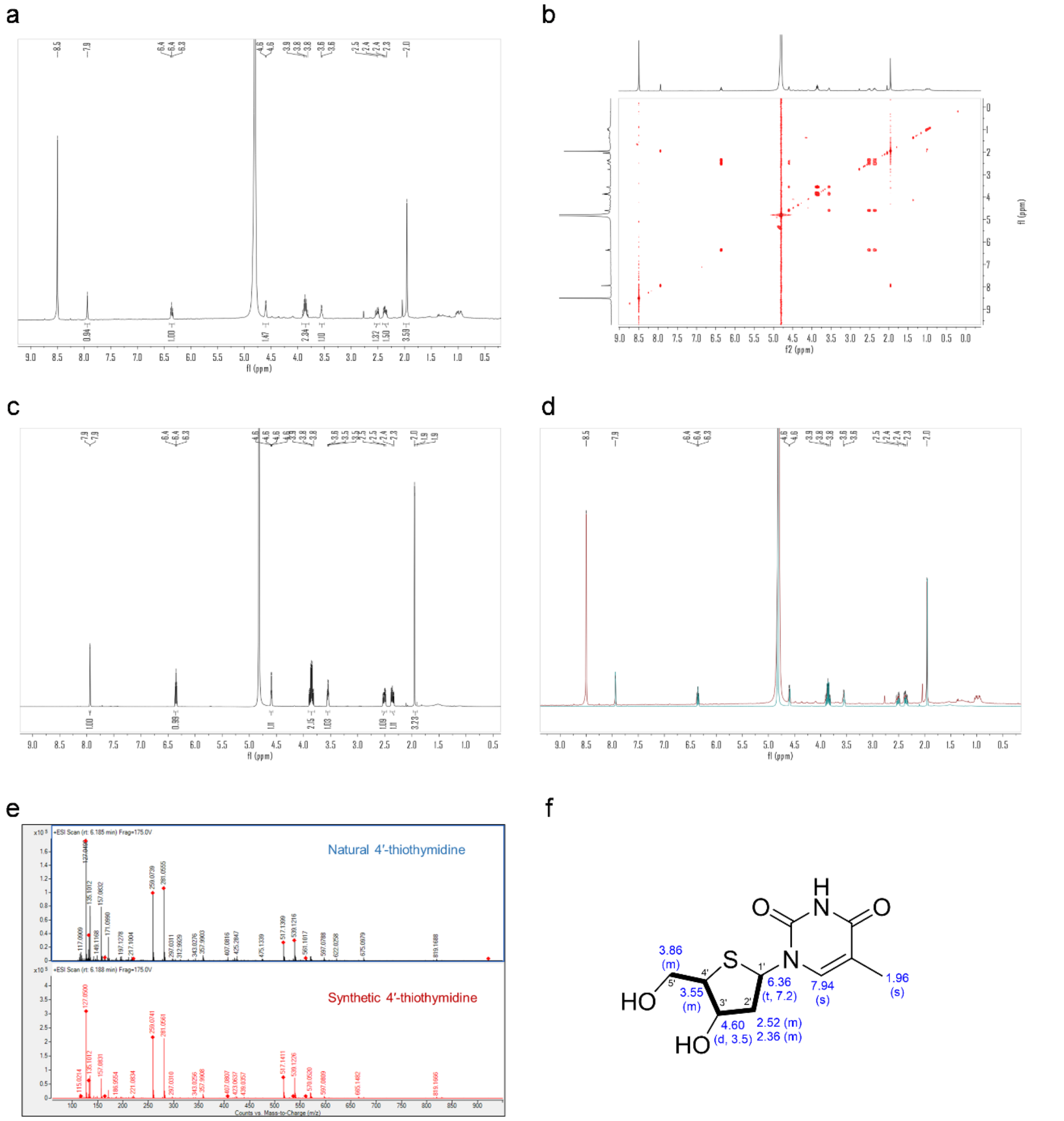

Figure S2. NMR spectroscopic and MS spectroscopic data of natural and synthetic 4′sT.
(a) ^{1}H NMR spectrum of natural 4′sT in D_2O (500 MHz).
(b) ^{1}H-^{1}H COSY NMR spectrum of natural 4′sT in D_2O (500 MHz).
(c) ^{1}H NMR spectrum of synthetic 4′sT in D_2O (500 MHz).
(d) Overlay of the ^{1}H NMR spectra of natural and synthetic 4′sT (500 MHz).
(e) MS spectra of natural (top) and synthetic (bottom) 4′sT.
(f) Chemical structure of 4′sT with assigned ^{1}H NMR chemical shifts (δ), multiplicity, and coupling constants (*J* in Hz). COSY correlations are indicated in bold.

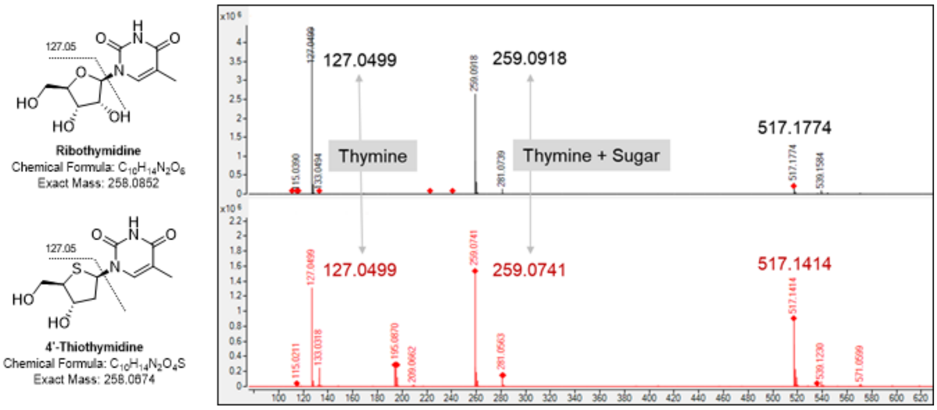

Figure S3. MS/MS spectra of ribothymidine and 4′-thiothymidine

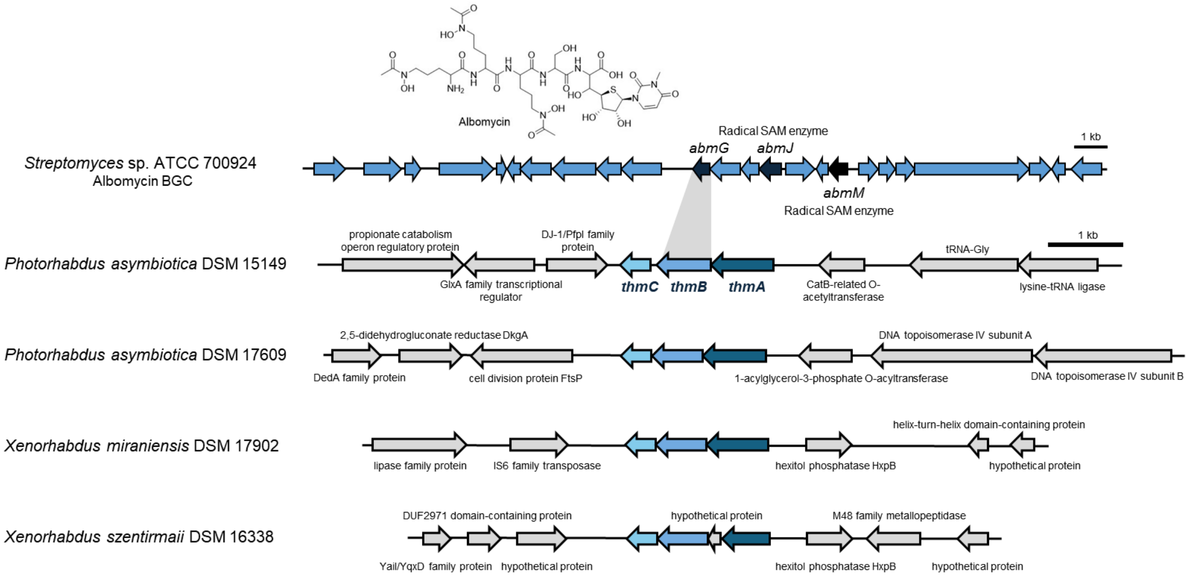

Figure S4. Identification of the 4′sT BGC. Homology-based search using genes from the albomycin BGC identified *thmB*, a distant homolog of *abmG*, in *P. asymbiotica* DSM 15149. This gene is clustered with a radical SAM enzyme (*thmA*) and a nucleoside triphosphatase (*thmC*). A cblaster search revealed that *thmA*-*C* co-occur as a conserved cluster across other *Photorhabdus* and *Xenorhabdus* genomes.

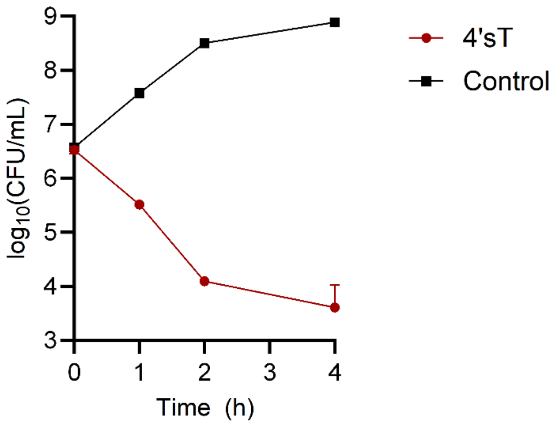

Figure S5. Time-kill assay. Exponential-phase cultures of *E. coli* MG1655 were treated with 16× MIC of 4′sT. Colony counts were determined at the indicated time points. Data represent mean ± s.d. from three independent replicates. CFU, colony-forming units.

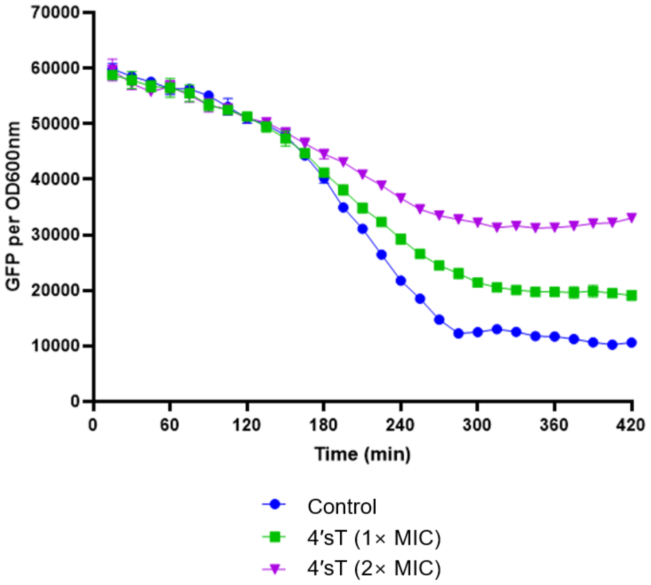

Figure S6. DNA supercoiling assay. MG1655-pSupR was grown in LB in a 96-well plate at 37 °C with or without 4′sT (green, 1× MIC; purple, 2× MIC). GFP fluorescence was measured every 15 min for 420 min. Curves represent the mean of three independent replicates.

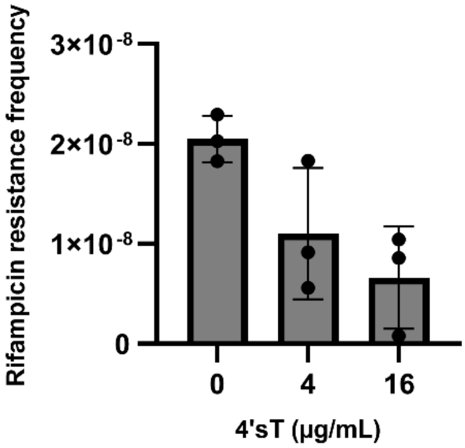

Figure S7. Effect of 4′sT on the frequency of antibiotic-resistant mutants. Overnight cultures of *E. coli* ATCC 25922 were diluted into MHIIB medium and incubated at 37°C with shaking at 220 rpm for 1.5 h before addition of 4′sT. After 4.5 h of total incubation, cultures were harvested, serially diluted, and plated to determine total CFU and rifampicin-resistant CFU per milliliter.

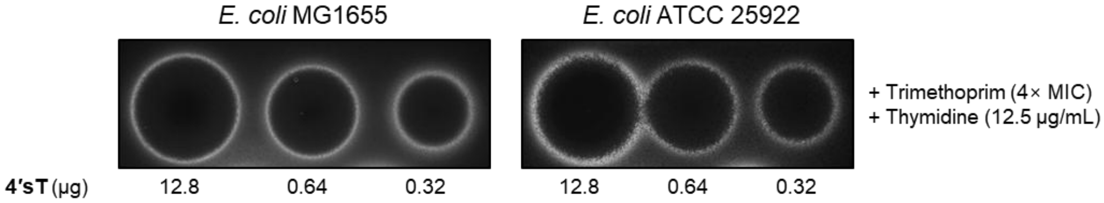

Figure S8. SOS response induction by 4′sT under conditions of impaired de novo thymidylate synthesis by trimethoprim.

Agar plate supplemented with trimethoprim (4× MIC) and thymidine (12.5 µg/mL) was overlaid with *E. coli* MG1655 and ATCC 25922 carrying the pDualrep2 (*sulA*::*rfp*) reporter plasmid and spotted with 4′sT. The plate was scanned using the Cy3 channel on a ChemiDoc system.
